# Genetic Diversity in the Modern Horse Illustrated from Genome-Wide SNP Data

**DOI:** 10.1371/journal.pone.0054997

**Published:** 2013-01-30

**Authors:** Jessica L. Petersen, James R. Mickelson, E. Gus Cothran, Lisa S. Andersson, Jeanette Axelsson, Ernie Bailey, Danika Bannasch, Matthew M. Binns, Alexandre S. Borges, Pieter Brama, Artur da Câmara Machado, Ottmar Distl, Michela Felicetti, Laura Fox-Clipsham, Kathryn T. Graves, Gérard Guérin, Bianca Haase, Telhisa Hasegawa, Karin Hemmann, Emmeline W. Hill, Tosso Leeb, Gabriella Lindgren, Hannes Lohi, Maria Susana Lopes, Beatrice A. McGivney, Sofia Mikko, Nicholas Orr, M. Cecilia T Penedo, Richard J. Piercy, Marja Raekallio, Stefan Rieder, Knut H. Røed, Maurizio Silvestrelli, June Swinburne, Teruaki Tozaki, Mark Vaudin, Claire M. Wade, Molly E. McCue

**Affiliations:** 1 University of Minnesota, College of Veterinary Medicine, St Paul, Minnesota, United States of America; 2 Texas A&M University, College of Veterinary Medicine and Biomedical Science, College Station, Texas, United States of America; 3 Swedish University of Agricultural Sciences, Department of Animal Breeding and Genetics, Uppsala, Sweden; 4 University of Kentucky, Department of Veterinary Science, Lexington, Kentucky, United States of America; 5 University of California Davis, School of Veterinary Medicine, Davis, California, United States of America; 6 Equine Analysis, Midway, Kentucky, United States of America; 7 University Estadual Paulista, Department of Veterinary Clinical Science, Botucatu-SP, Brazil; 8 University College Dublin, School of Veterinary Medicine, Dublin, Ireland; 9 University of Azores, Institute for Biotechnology and Bioengineering, Biotechnology Centre of Azores, Angra do Heroísmo, Portugal; 10 University of Veterinary Medicine Hannover, Institute for Animal Breeding and Genetics, Hannover, Germany; 11 University of Perugia, Faculty of Veterinary Medicine, Perugia, Italy; 12 Animal Health Trust, Lanwades Park, Newmarket, Suffolk, United Kingdom; 13 French National Institute for Agricultural Research-Animal Genetics and Integrative Biology Unit, Jouy en Josas, France; 14 University of Sydney, Veterinary Science, New South Wales, Australia; 15 Nihon Bioresource College, Koga, Ibaraki, Japan; 16 University of Helsinki, Faculty of Veterinary Medicine, Helsinki, Finland; 17 University College Dublin, College of Agriculture, Food Science and Veterinary Medicine, Belfield, Dublin, Ireland; 18 University of Bern, Institute of Genetics, Bern, Switzerland; 19 Institute of Cancer Research, Breakthrough Breast Cancer Research Centre, London, United Kingdom; 20 Royal Veterinary College, Comparative Neuromuscular Diseases Laboratory, London, United Kingdom; 21 Swiss National Stud Farm, Agroscope Liebefeld-Posieux Research Station, Avenches, Switzerland; 22 Norwegian School of Veterinary Science, Department of Basic Sciences and Aquatic Medicine, Oslo, Norway; 23 Animal DNA Diagnostics Ltd, Cambridge, United Kingdom; 24 Laboratory of Racing Chemistry, Department of Molecular Genetics, Utsunomiya, Tochigi, Japan; University of Uppsala, Sweden

## Abstract

Horses were domesticated from the Eurasian steppes 5,000–6,000 years ago. Since then, the use of horses for transportation, warfare, and agriculture, as well as selection for desired traits and fitness, has resulted in diverse populations distributed across the world, many of which have become or are in the process of becoming formally organized into closed, breeding populations (breeds). This report describes the use of a genome-wide set of autosomal SNPs and 814 horses from 36 breeds to provide the first detailed description of equine breed diversity. F_ST_ calculations, parsimony, and distance analysis demonstrated relationships among the breeds that largely reflect geographic origins and known breed histories. Low levels of population divergence were observed between breeds that are relatively early on in the process of breed development, and between those with high levels of within-breed diversity, whether due to large population size, ongoing outcrossing, or large within-breed phenotypic diversity. Populations with low within-breed diversity included those which have experienced population bottlenecks, have been under intense selective pressure, or are closed populations with long breed histories. These results provide new insights into the relationships among and the diversity within breeds of horses. In addition these results will facilitate future genome-wide association studies and investigations into genomic targets of selection.

## Introduction

With a world-wide population greater than 58 million [1], and as many as 500 different breeds, horses are economically important and popular animals for agriculture, transportation, and recreation. The diversity of the modern horse has its roots in the process of domestication which began 5,000–6,000 years ago in the Eurasian Steppe [2]–[4]. Unlike other agricultural species such as sheep [5] and pigs [6], [7], archaeological and genetic evidence suggests that multiple horse domestication events occurred across Eurasia [2], [8]–[12]. During the domestication process, it is believed that gene flow continued between domesticated and wild horses [13] as is likely to also have been the case during domestication of cattle [14], [15]. Concurrent gene flow between domestic and wild horses would be expected to allow newly domestic stock to maintain a larger extent of genetic diversity than if domestication occurred in one or few events with limited individuals.

Prior genetic work aimed at understanding horse domestication has shown that a significant proportion of the diversity observed in modern maternal lineages was present at the time of domestication [2], [8], [16]. The question of mitochondrial DNA (mtDNA) diversity was further addressed by recent sequencing of the entire mtDNA genome. These studies estimate that, minimally, 17 to 46 maternal lineages were used in the founding of the modern horse [2], [17]; however, those data were unable to support prior studies suggesting geographic structure among maternal lineages [9], [18]. Recent nuclear DNA analyses have utilized “non-breed” horses sampled across Eurasia to attempt to understand the population history of the horse. These microsatellite-based studies suggest a weak pattern of isolation by distance with higher levels of diversity in, and population expansion originating from Eastern Asia [13], [19]. High diversity as observed by both mtDNA and microsatellites and the absence of strong geographical patterns is likely a result of continued gene flow during domestication, the high mobility of the horse, and its prevalent use for transportation during and after the time of domestication. Interestingly, while significant diversity is observed in maternal lineages, paternal input into modern horse breeds appears to have been extremely limited as shown by a lack of variation at the Y-chromosome [20], [21].

Diversity in the founding population of the domestic horse has since been exploited to develop a wealth of specialized populations or breeds. While some breeds have been experiencing artificial selection for hundreds of years (e.g. Thoroughbred, Arabian), in general, most modern horse breeds have been developed recently (e.g. Quarter Horse, Paint, Tennessee Walking Horse) and continue to evolve based upon selective pressures for performance and phenotype (Table 1). Horse breeds resulting from these evolutionary processes are generally closed populations consisting of individual animals demonstrating specific phenotypes and/or bloodlines. Each breed is governed by an independent set of regulations dictated by the respective breed association. Not all breeds are closed populations. Some breed registries allow admixture from outside breeds (e.g. Swiss Warmblood, Quarter Horse), and others are defined by phenotype (e.g. Miniature). Finally, some populations that are often referred to as breeds are classified simply by their geographic region of origin and may not be actively maintained by a formal registry (e.g. Mongolian, Tuva) (Table 1). Those breeds that may be free ranging and experience lesser degrees of management may more appropriately be termed “landrace populations.” Therefore, genetic characteristics within horse breeds are expected to differ based upon differences in the definition of the breed, the diversity of founding stock, the time since breed establishment, and the selective pressures invoked by breeders. The extent of gene flow not only varies within breed, but among horse breeds, the direction and level of gene flow is influenced by breed restrictions/requirements, and potentially by geographic distance.

**Table 1:** Populations (breeds) included in the study, region of breed origin and sampling location, notes on population history relevant to diversity statistics, and breed classification based upon use and phenotype.

Breed	Geographic Origin	Region Sampled	Population size (approx)	Population Notes	Classification(s)
Akhal Teke	Turkmenistan	US & Russia	3,500	Pedigree records began-1885, Stud book-1941	Riding horse, endurance
Andalusian	Spain	United States	185,000	US registry formed in 1995 including Pura Raza Española & Lusitano bloodlines	Riding horse, sport
Arabian	Middle East	United States	1 million	Arabian type bred for over 3,500 years; US stud book-1908	Riding horse, endurance
Belgian	Belgium	United States	common	US Association began-1887	Draft
Caspian	Persia	United States	rare	Rediscovered in 1965 with N∼50, no breeding records prior; Stud book-1966	Riding and driving pony
Clydesdale	Scotland	US & UK	5,000	Registry formed-1877 in Scotland; Stud book-1879	Draft
Exmoor	Great Britain	UnitedKingdom	2,000	Exmoor Pony Society-1921	Riding and driving pony
Fell Pony	England	UnitedKingdom	6,000	Fell Pony Society began in 1922; outcrossed with Dale’spony until 1970s	Light draft pony
Finnhorse	Finland	Finland	19,800	Stud book-1907	Light draft; riding horse; trotting
FloridaCracker	United States	United States	rare	Introduced to US in 1500s; association began-1989 with 31 horses	Riding horse, gaited
Franches-Montagnes	Switzerland	Switzerland	21,000	Official stud book-1921; Current breeding association established-1997	Light draft, riding horse
French Trotter	France	France	common	Population closed-1937 although allows some Standardbred influence	Riding horse, trotting
Hanoverian	Germany	Germany	20,000 (Germany)	Outcrossing allowed	Riding horse
Icelandic	Iceland	Sweden	180,000	Isolated >1,000 years; Federation of Icelandic HorseAssociation began-1969	Riding horse, gaited
Lusitano	Portugal	Portugal	12,000	Stud book-1967 after split from Spanish Andalusian breed	Riding horse, sport
MangalargaPaulista	Brazil	Brazil	common	Registry began-1934	Riding horse
Maremmano	Italy	Italy	7,000	Breed identification based upon conformation andinspection	Riding horse
Miniature	United States	United States	185,000	Two US registries founded in 1970s; Maximum height restrictions for registration	Driving pony, extreme small size
Mongolian	Mongolia	Mongolia	2 million	Many types based upon purpose and geography	Riding horse, landrace
Morgan	United States	United States	100,000	Founding sire born in 1789; Registry-1894	Riding and driving horse
New ForestPony	England	UnitedKingdom	15,000	Stud book-1910 with a variety of sires; No outcrossingsince 1930s	Light draft, riding pony, landrace
North SwedishHorse	Sweden	Sweden	10,000	Breed association-1894; Stud book-1915	Draft
NorwegianFjord	Norway	Norway	common	Stud book-1909	Riding and light draft
Paint	United States	United States	1 million	Registry-1965; One parent can be Quarter Horse or Thoroughbred	Riding horse, stock horse
Percheron	France	United States	20,000	Stud book-1893	Draft
PeruvianPaso	Peru	United States	25,000	Breed type over 400 years old; Closed population	Riding horse, gaited
Puerto RicanPaso Fino	Puerto Rico	Puerto Rico	250,000	Breed type ∼500 years old; Association founded-1972	Riding horse, gaited
Quarter Horse	United States	United States	4 million	Association formed-1940; One parent may be Paint or Thoroughbred	Riding horse, stock horse, racing
Saddlebred	United States	United States	75,000	Breed type founded in late 1700s; Association began-1891	Riding and driving horse, some gaited
Shetland	Scotland	Sweden	common	Stud book-1891	Riding pony
Shire	England	United States	7,000	1st Shire organization-1877 (UK); stud book-1880;US assoc-1885	Draft
Standardbred	United States	Norway	common	Stud book-1871; Some outside trotting bloodlines(French Trotter) allowed	Riding horse, harness racing (trot)
Standardbred	United States	United States		Stud book-1871; Harness racing in early 1800s includedpacing horses	Riding horse, harness racing (trot or pace)
Swiss Warmblood	Switzerland	Switzerland	15,000	Stud book-1921; Crossed with European Warmbloods, Thoroughbreds, Arabians	Riding horse, sport
Tenn Walking Horse	United States	United States	500,000	Registry-1935; Blood typing and parentage verification mandated in 1993	Riding horse, gaited
Thoroughbred	England	UK & Ireland	common	Stud book-1791; Closed population	Race horse, riding horse, sport
Thoroughbred	England	United States			Race horse, riding horse, sport
Tuva	Siberia	Russia	30,000	Different types depending on region	Light draft, landrace

Considering modern breeds, unlike mtDNA, nuclear markers can discern breed membership [12]. However, studies of nuclear genetic diversity of modern breeds to date have most commonly focused on a single population of interest, sets of historically related breeds, or breeds within a specific geographic region [22]–[36]. Additionally, these analyses of nuclear genetic diversity in horse breeds are largely based upon microsatellite loci, which do not often permit consolidation of data across studies. Thus, large, across-breed investigations of nuclear diversity in the modern, domestic horse are lacking.

The Equine Genetic Diversity Consortium (EGDC), an international collaboration of the equine scientific community, was established in an effort to quantify nuclear diversity and the relationships within and among horse populations on a genome-wide scale. The development of this consortium has facilitated the collection of samples from 36 breeds for genotyping on the Illumina 50K SNP Beadchip. The breeds included in this report represent many of the most popular breeds in the world as well as divergent phenotypic classes, different geographic regions of derivation, and varying histories of breed origin (Table 1). The standardized SNP genotyping platform permits the compilation of data across breeds at a level never before achieved. Results of this collaboration now allow for the detailed description of diversity and assessment of the effects of genetic isolation, inbreeding, and selection within breeds, and the description of relationships among breeds. These data will also facilitate future across breed genome-wide association studies as well as investigations into genomic targets of selection.

## Results

### Samples

Of the 38 populations sampled, two breeds were represented by geographically distinct populations: the Thoroughbred was sampled in the both the United States (US) and the United Kingdom and Ireland (UK/Ire), and the Standardbred was sampled in the US as well as in Norway. Eight Standardbred horses sampled from the US were noted to be pacing horses as opposed to the Norwegian and remaining US individuals that were classified as trotters. In addition, the International Andalusian and Lusitano Horse Association Registry (IALHA) in the US maintains one stud book but designates whether the individual was derived from Spanish (Pura Raza Española) or Portuguese (Lusitano) bloodlines, or a combination of both. Of the Andalusian horses collected in the US, five were noted to have Portuguese bloodlines.

Phenotypic classifications of the horse breeds include those characterized by small stature (Miniature Horse, pony breeds), breeds characterized by large stature and/or large muscle mass in proportion to size (draft breeds), light horse or riding breeds, gaited breeds, rare breeds, breeds founded in the past 80 years, and populations that are relatively unmanaged (“landrace”). The number of samples, sampling location, region of breed origin, and a list of primary breed characteristics are found in Table 1.

After pruning of individuals for genotyping quality and relationships (see methods), and keeping a similar number of individuals per breed, 814 of the 1,060 horses remained in the analysis. Of the horses removed, 12 had known pedigree relationships at or more recent to the grandsire/dam level, 44 individuals were removed at random from overrepresented breeds to equalize sample size across breeds, 4 failed to genotype at a rate greater than 0.90, and 186 were removed due to pi hat values (pairwise estimates of identity by descent) above the allowed threshold. Of those last 186 horses that were removed, 122 were from disease studies where relationships were common due to sampling bias.

### Within Breed Diversity

Diversity indices were calculated using 10,536 autosomal SNPs that remained after pruning for minor allele frequency (MAF), genotyping rate, and linkage disequilibrium (LD) across breeds (referred to as the primary SNP set). Diversity indices were also calculated using three other SNP sets, resulting from different levels of LD-based pruning (see methods). Individuals noted as outliers in parsimony and cluster analyses (see below) were excluded from within-breed diversity calculations.

Using the primary SNP set, diversity, as measured by expected heterozygosity (H_e_), ranged from 0.232 in the Clydesdale, to 0.311 in the Tuva (Table 2). Considering the SNP sets pruned less stringently for LD, the diversity within the Thoroughbred increased in relationship to the other breeds, as did that of nine other breeds. Mean and total heterozygosity increased with increased number of loci and less stringent LD pruning (Table 2). Inbreeding coefficients (F_IS_) calculated on the primary SNP set showed significant excess homozygosity in 17 populations, which was greatest in the Andalusian (0.065). Three of the four lowest F_IS_ values were found in the Thoroughbred samples (UK/Ire, US, and when considered together) (Table 2).

**Table 2:** Number of samples (N), effective population size (N_e_), individual inbreeding estimates (f), inbreeding coefficient (F_IS_), and expected heterozygosity (H_e_) from four SNP sets pruned based upon varying levels of LD.

							Expected Heterozygosity (He)			
				Individual inbreeding (f)			r2 0.1	R2 0.1	r2 0.2	r2 0.4
Breed	N	Ne	FIS	Min	Max	Mean	10,536	6,028	18,539	26,171
Akhal Teke	19	302	0.015*	0.015	0.297	0.101	0.287	0.281	0.303	0.311
Andalusian	18a	329	0.065*	0.028	0.274	0.114	0.296	0.293	0.308	0.312
Arabian	24a	346	0.033*	0.060	0.060	0.060	0.287	0.280	0.302	0.310
Belgian	30b	431	−0.002	0.039	0.166	0.111	0.278	0.276	0.284	0.284
Caspian	18	351	−0.022	−0.033	0.136	0.041	0.294	0.292	0.305	0.308
Clydesdale	24	194	0.004	0.128	0.323	0.261	0.232	0.225	0.238	0.236
Exmoor	24	216	0.034*	0.055	0.556	0.239	0.247	0.242	0.253	0.252
Fell Pony	21	289	0.002	0.069	0.178	0.114	0.278	0.272	0.285	0.285
Finnhorse	27	575	−0.004	0.011	0.100	0.052	0.296	0.296	0.302	0.301
Florida Cracker	7	171	0.026*	0.004	0.359	0.159	0.270	0.263	0.284	0.291
Franches-Montagnes	19a	316	0.003	0.018	0.203	0.095	0.284	0.279	0.297	0.301
French Trotter	17a	233	−0.018	0.064	0.173	0.105	0.275	0.262	0.295	0.307
Hanoverian	15a	269	−0.010	0.002	0.087	0.052	0.294	0.280	0.320	0.335
Icelandic	25c	555	0.006*	0.043	0.234	0.083	0.289	0.288	0.290	0.288
Lusitano	24	391	0.039*	0.008	0.220	0.090	0.296	0.292	0.309	0.315
Maremmano	24	341	−0.012	−0.015	0.109	0.038	0.298	0.287	0.318	0.329
Miniature	21	521	0.005	0.043	0.161	0.075	0.291	0.292	0.296	0.295
Mangalarga Paulista	15	155	−0.011	0.176	0.320	0.242	0.235	0.228	0.246	0.250
Mongolian	19a	751	0.001	−0.034	0.055	0.015	0.309	0.308	0.314	0.314
Morgan	40	448	0.040*	0.003	0.307	0.090	0.296	0.287	0.310	0.317
New Forest Pony	15	474	0.000	−0.022	0.066	0.025	0.304	0.300	0.316	0.319
Norwegian Fjord	21a	335	−0.003	0.053	0.168	0.122	0.274	0.274	0.278	0.277
North Swedish Horse	19	369	0.011*	0.069	0.210	0.133	0.275	0.276	0.279	0.278
Percheron	23	451	0.003	0.043	0.143	0.086	0.287	0.284	0.292	0.293
Peruvian Paso	21	433	0.002	0.008	0.134	0.055	0.298	0.293	0.306	0.310
Puerto Rican Paso Fino	20	321	−0.003	0.004	0.298	0.103	0.280	0.278	0.287	0.290
Paint	25	399	0.006*	−0.013	0.101	0.040	0.302	0.289	0.324	0.337
Quarter Horse	40a	426	0.011*	−0.012	0.144	0.047	0.302	0.290	0.323	0.336
Saddlebred	25d	297	−0.008	0.051	0.145	0.103	0.279	0.268	0.297	0.306
Shetland	27	365	0.032*	0.108	0.370	0.182	0.264	0.268	0.268	0.266
Shire	23	357	0.024*	0.130	0.258	0.187	0.261	0.252	0.268	0.267
Standardbred - Norway	25e	232	−0.004	0.063	0.202	0.130	0.272	0.255	0.289	0.298
Standardbred - US	15	179	0.039*	0.097	0.222	0.153	0.276	0.262	0.293	0.303
Standardbred - all	40	290	0.022*	−0.028	0.323	0.130	0.276	0.260	0.293	0.303
Swiss Warmblood	15a	271	0.005	0.023	0.117	0.059	0.296	0.281	0.322	0.337
Thoroughbred - UK/Ire	19a	143	−0.028	0.089	0.171	0.133	0.264	0.245	0.292	0.309
Thoroughbred - US	17a	163	−0.015	0.093	0.182	0.134	0.267	0.250	0.295	0.313
Thoroughbred - all	36	190	−0.019	0.089	0.182	0.134	0.266	0.248	0.294	0.312
Tuva	15	533	0.016*	−0.028	0.116	0.022	0.311	0.309	0.320	0.322
Tennessee Walking Horse	19	230	0.008*	0.065	0.276	0.148	0.269	0.256	0.284	0.291
Mean	22.3	341	0.007	0.039	0.204	0.107	0.282	0.275	0.295	0.300
Total	814						0.313	0.303	0.329	0.336
Min			−0.028	−0.034	0.055	0.015	0.232	0.225	0.238	0.236
Max			0.005	0.176	0.556	0.261	0.311	0.309	0.324	0.337

a Individuals from this breed also included in [41];

b 20 of these individuals were also reported in [41];

c 17 of these individuals were also reported in [41];

d 21 of these individuals were also reported in [41];

e 19 of these individuals were also reported in [41].

F_IS_ and f were calculated based upon the primary SNP set (10,536 loci). Samples also used in [41] are indicated in the footnotes.

* indicates significance at α<0.05 determined by 10,000 permutations.

Inbreeding coefficients (f) calculated for each individual based upon observed and expected heterozygosity showed several individuals with significant loss of heterozygosity. The highest individual value of f (0.56) was found in an Exmoor pony. Within breeds, average individual estimates of f were greatest in the Clydesdale, Mangalarga Paulista, and Exmoor while the lowest breed means were found in the landrace populations (Table 2).

Effective population size (N_e_), as estimated by LD [37] using an autosomal SNP set pruned within each breed for quality, was lowest (143) in the UK/Ire sample of the Thoroughbred (UK/Ire) but also low in the other racing breeds as well as the Clydesdale (Table 2). Highest values of N_e_ were observed in the Eurasian landrace populations, the Mongolian (743) and Tuva (533), and also in the Icelandic (555), Finnhorse (575), and Miniature (521). Breed-specific decay of LD essentially mirrors the results of the N_e_ calculation given the relationship between the statistics. A plot of LD across 2 Mb in a subset of the breeds that represent the range of N_e_ estimates is found in Figure S1.

### Parsimony and Principal Component Analyses

**Figure 1:**
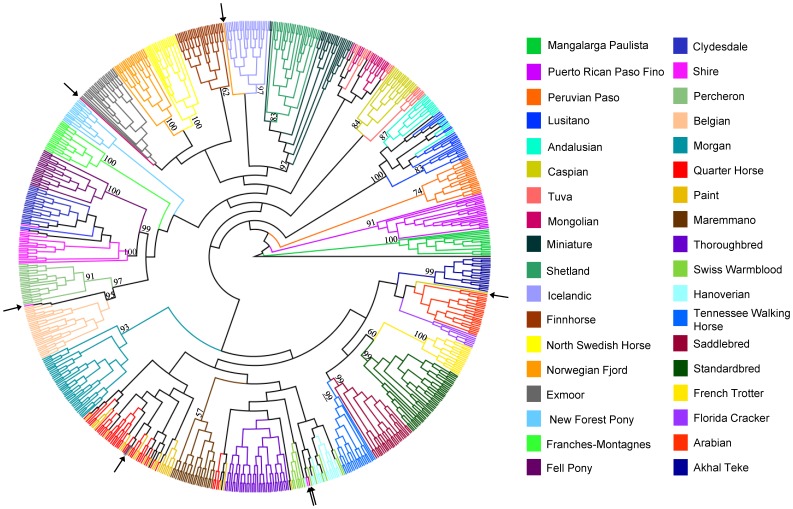
Individual and breed relationships among 814 horses illustrated by parsimony. Parsimony tree created from 10,066 SNPs and rooted by the domestic ass. Breeds are listed in the legend in order starting from the root and working counterclockwise. Individual outliers with respect to their breeds are noted with arrows. Bootstrap support calculated from 1,000 replicates is shown for major branches when greater than 50%.

With a domestic ass designated as the outgroup, parsimony analysis of 10,066 loci pruned for LD of R^2^ = 0.2 (see methods) resulted in generally tight clustering and monophyly of samples within breeds, supported by high bootstrap values (Figure 1). Major clades of the tree show grouping of the Iberian breeds (Lusitano and Andalusian), ponies (Icelandic, Shetland, Miniature), Scandinavian breeds (Finnhorse, North Swedish Horse, Norwegian Fjord), heavy draft horses (Clydesdale, Shire, Belgian, Percheron), breeds recently admixed with and/or partly derived from the Thoroughbred (Paint, Quarter Horse, Maremmano, Swiss Warmblood, Hanoverian), modern US breeds (American Saddlebred (hereafter “Saddlebred” and Tennessee Walking Horse), trotting breeds (Standardbred and French Trotter), and Middle Eastern breeds (Akhal Teke and Arabian). Exceptions to monophyly include the Paint and Quarter Horse as well as the Hanoverian and Swiss Warmblood, which are mixed in clades surrounding the Thoroughbred and Maremmano. In addition, the Clydesdale was placed as a clade within the Shire breed and the Shetlands as a clade within the Miniatures. Strong bootstrap support for monophyly is present within a subset each of Lusitanos (83%), and Andalusians (87%); however the remainder of individuals from these breeds were intermixed. No structure was found within the US sample regarding individual Andalusians noted to have Portuguese bloodlines opposed to those with Spanish bloodlines (Figure S2). The Mongolian and most Tuva horses were grouped together while a subset of the Tuvas fell out as a sister clade to the Caspians. Several individuals were not positioned in the clades that represented the majority of the other individuals in the breed (Figure 1). These include three Shires, two Mongolians, a Caspian, and a Norwegian Fjord. In each instance, the outlier status of these individuals was also supported by cluster analysis (see below).

Principal component analysis (PCA) also serves to visualize individual relationships within and among breeds. The plot of PC1 vs. PC2 shown in Figure S3 illustrates relationships similar to those shown by parsimony, including the placement of outliers outside of their respective breeds. All Thoroughbred samples, regardless of origin, are separated from the others by PC1 and form a cluster at the top of the figure. Intermediate between the Thoroughbred and central cluster of breeds are the Hanoverian, Swiss Warmblood, Paint, and Quarter Horse. The Shetland, Icelandic, and Miniature split from the remainder of samples in PC2, falling out in the lower left corner, and the British drafts anchor the figure at the lower right. While most breeds cluster tightly, several are dispersed across one or both PCs. The Hanoverian, Swiss Warmblood, Paint, and Quarter Horse, as noted above, are extended along PC1, while the Arabian and Franches-Montagnes show similar spreading, also along PC1. The Tuva, Clydesdale, and Shire individuals also are not as tightly clustered as other populations despite the low within breed diversity of the latter two.

### Distance Analysis

**Figure 2:**
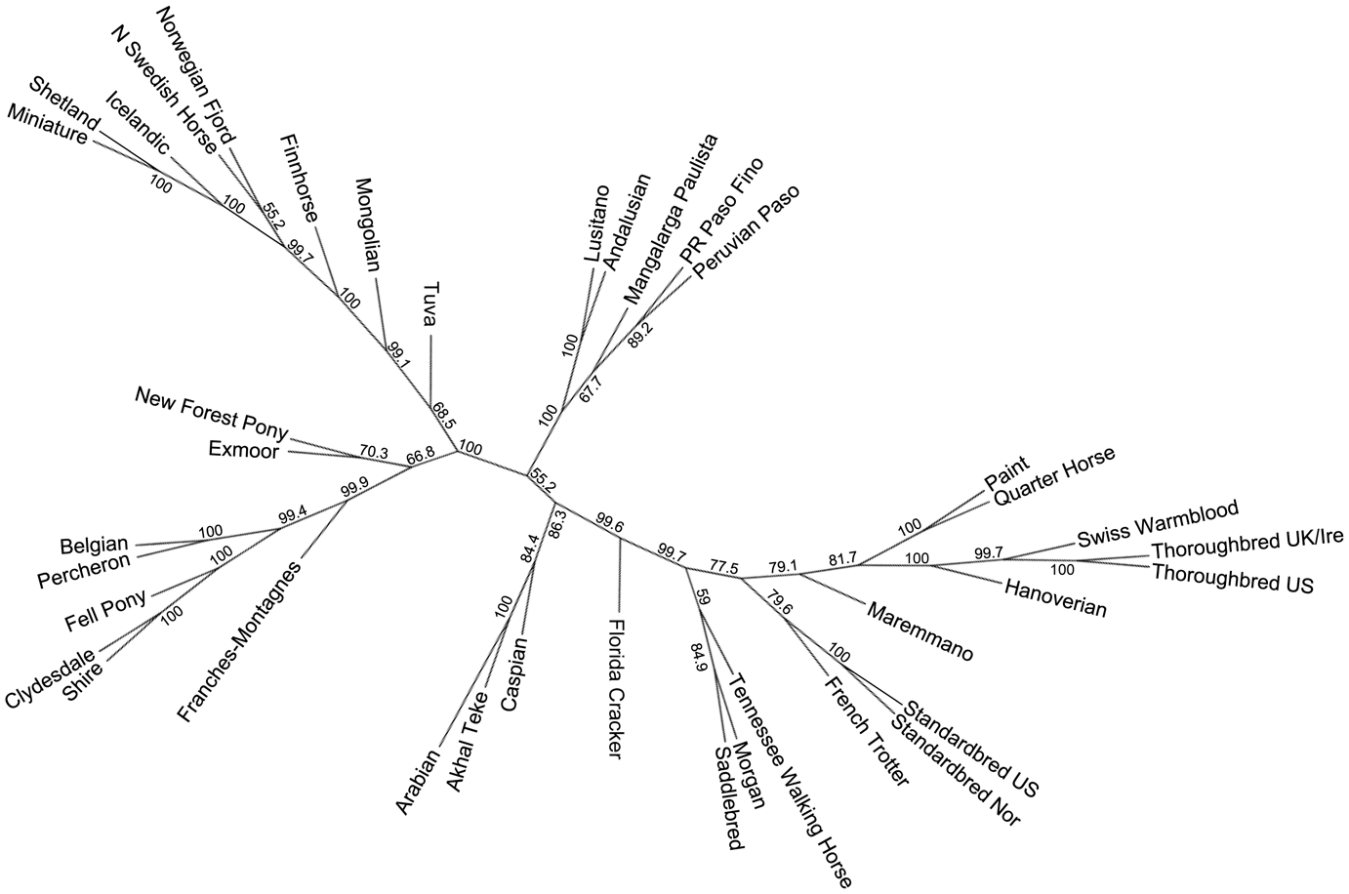
Distance based, neighbor joining tree calculated from SNP frequencies in 38 horse populations. Majority rule, neighbor joining tree created from 10,536 SNP makers using Nei’s genetic distance and allele frequencies within each population. Percent bootstrap support for all branches calculated from 1,000 replicates is shown.

An unrooted neighbor joining (NJ) tree of Nei’s distance [38] was constructed using SNP frequencies within breeds from the 10,536 SNP data set (Figure 2). The relative placement of breeds reflects that seen in the parsimony tree with several exceptions. The Paint, Quarter Horse, Swiss Warmblood, Hanoverian, Maremmano, and Thoroughbred, are found in one large branch of the tree, although the Maremmano is placed outside of the clade containing the aforementioned breeds. The position of the Morgan with the Saddlebred and Tennessee Walking Horse also deviates from parsimony analysis but reflects historic records of relationships among these breeds. The Scandinavian breeds remain in one branch of the clade, which also includes the Shetland and Miniature. Unlike the parsimony cladogram, the Caspian falls in a clade with the other Middle Eastern breeds, the Arabian and Akhal Teke. Finally, the Exmoor, a British breed, is placed with another British breed, the New Forest Pony, rather than with the Scandinavian breeds as in the parsimony analysis. Each branch shows support of over 50%, with many clades being supported by over 99% of the 1,000 bootstrap replicates.

### Cluster analysis

Likelihood scores for runs of various K in Structure showed an increase in overall mean ln P(X|K) until K = 35 (Figure S4). A clear “true” value of K is not obvious examining the likelihood scores or using the Evanno method [39] (data not shown); however, variance among runs begins to increase with a diminishing increase in likelihood scores after K = 29, which is near the peak of the curve. The value of the highest proportion (breed average q-value) of assignment of each breed for each value of K, as well as the cluster to which it assigns is shown in Table S1. Additionally, the proportion assignment at K = 29 for each of the breeds is found in Table S2.

**Figure 3:**
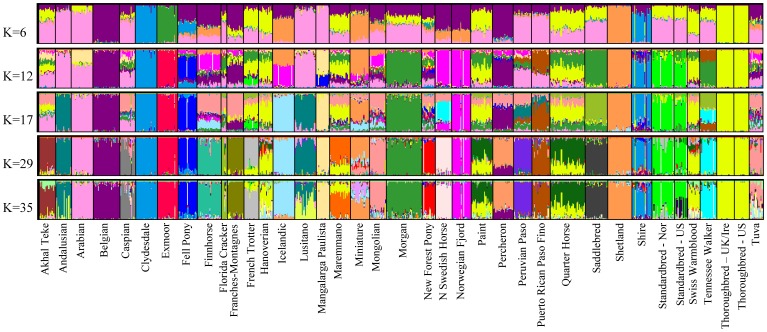
Bayesian clustering output for five values of K in 814 horses of 38 populations. Structure output for five values of K investigated. Each individual is represented by one vertical line with the proportion of assignment to each cluster shown on the y axis and colored by cluster. Other values of K are shown in Figure S1 and a summary of assignment of each breed in Tables S1 and S2.

The first breeds to have all individuals assign strongly to one cluster are the Thoroughbred and Clydesdale (with Shire) at K = 2, followed by the Shetland at K = 3; these four breeds do not show signs of admixture at any K value analyzed. Evidence of weak geographic grouping is observed at K  = 4, which consists of: 1, the Middle Eastern and Iberian breeds (pink); 2, the Thoroughbred and breeds to which it continues to be or was historically crossed (yellow); 3, breeds developed in Scandinavia and Northern Europe (orange); and 4, the British Isles draft breeds (blue) (Figure 3).

### Middle Eastern and Iberian Breeds

As also observed in the NJ tree, clustering of the Iberian and Middle Eastern breeds with the Mangalarga Paulista, Peruvian Paso, and Puerto Rican Paso Fino (q >0.5) is observed until K  = 8, after which point the Mangalarga Paulista assigns with q  = 0.93 to another cluster. The remaining breeds cluster together until K = 12, at which time the Middle Eastern breeds (Arabian, Akhal Teke, and Caspian) are assigned to their own cluster, leaving the Iberian breeds clustered with the Peruvian Paso and Puerto Rican Paso Fino. At low values of K (*i.e.* K <6) the Florida Cracker, Saddlebred, Standardbreds, Morgan, and Tennessee Walking Horse fall into the cluster with the Iberian and Middle Eastern breeds with breed mean q >0.5. At K = 29, each of these breeds is assigned with q >0.72 to an individual cluster with the exception of the Lusitano and Andalusian, which remaining clustered together.

### Thoroughbreds and Thoroughbred Crossed Breeds

Relationships described by the NJ tree among the Thoroughbred, Hanoverian, Swiss Warmblood, Paint, Quarter Horse, and Maremmano are also seen in cluster analysis. Clustering of those breeds with the Thoroughbred is observed throughout the values of K examined although at moderate frequencies (Figure 3, Table S1, Figure S5). At K  = 29, the Hanoverian and Swiss Warmblood remain assigned to the cluster defined by the Thoroughbred but with assignment probabilities of 0.51 each. The Quarter Horse and Paint also assign to this cluster with q-values of 0.30 and 0.34, respectively. Neither the Quarter Horse, Paint, Hanoverian, or Swiss Warmblood populations assign to any cluster with q >0.62 at K  = 29. No evidence of population substructure is observed between the US and UK/Ire Thoroughbreds as also shown by PCA and parsimony analyses (Figure S6).

### Scandinavian and Northern European Breeds

As in the NJ and parsimony trees, the Finnhorse, Icelandic, Miniature, North Swedish Horse, Norwegian Fjord, and Shetland are parsed into the same cluster (q-value >0.5) through K  = 5. However, unlike the NJ tree, at K  = 4, the highest value of assignment places the Belgian and Percheron into this cluster although with q <0.5 (0.42 and 0.38, respectively). The relationship remains until K  = 6, at which time the Miniature, Icelandic, and Shetland fall into a different cluster. At K  = 10, the Icelandic clusters again with the North Swedish Horse and Norwegian Fjord. The Norwegian and United States Standardbred populations, which at K  = 4 assign with q >0.5 to the cluster containing the Scandinavian breeds, separate from the Scandinavian breeds at K  = 5. At K  = 31, substructure appears in the Standardbred samples, which correlates to those individuals identified as pacers and that fall into an individual clade in the parsimony tree (Figure S7). At K  = 29, the Miniature and Shetland continue to be assigned to the same cluster (q-values  = 0.55 and 0.95, respectively). The next highest proportions of assignment of the Miniature horse are to the clusters described by the New Forest Pony (q  = 0.20) and Icelandic (q  = 0.11). No value of K evaluated eliminated signals of admixture from all populations in the dataset at K = 38 (the actual number of populations sampled) or any value of K through 45 (data not shown).

### British Isles Draft

The Clydesdale and Shire cluster together, and apart from the other breeds beginning at K  = 3. In addition, the Fell Pony, which is placed within the same clade in the NJ and parsimony trees, and proximal to the Clydesdale and Shire in PCA, shows moderate assignment to this cluster (0.29< q <0.41) for several values of K from 4 to 14. At K  = 29, the Shire assigns to the same cluster as the Clydesdale with q  = 0.69. The individual outliers from the Shire breed also noted in parsimony analysis are evident beginning at K  = 3. Excluding these outliers, at K  = 29, the proportion of assignment for the Shires to the cluster with the Clydesdale increases to 0.74.

### F_ST_

**Figure 4:**
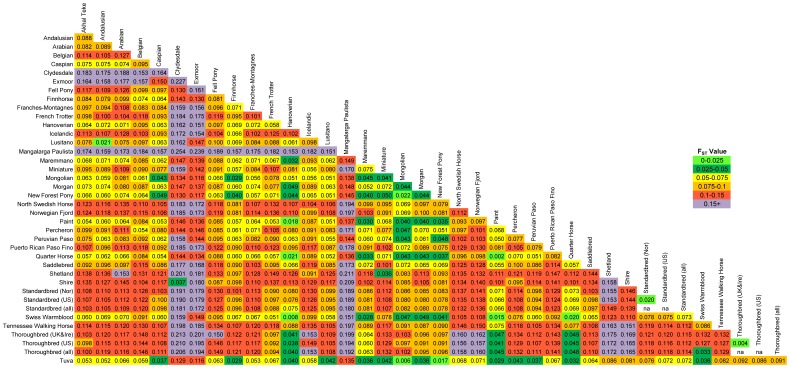
Pairwise F_ST_ values based upon 10,536 SNPs in 37 horse populations. Pairwise F_ST_ values as calculated in Arlequin using 10,536 autosomal SNPs and significance tested using 20,000 permutations. All pairwise values are significantly different from zero. (individual outliers were removed from this analysis).

All pairwise F_ST_ values calculated between the 37 populations (excluding the Florida Cracker) were significant as tested by 20,000 permutations (Figure 4). The lowest level of differentiation was found between the Paint and Quarter Horse populations (F_ST_  = 0.002), while the greatest divergence was observed between the Clydesdale and Mangalarga Paulista (F_ST_ = 0.254). The two Thoroughbred populations had an F_ST_ value of 0.004, while the two Standardbred populations had 10-fold greater divergence (F_ST_  = 0.020) than the minimum observed value in this dataset; this value is similar to that observed between the Lusitano and Andalusian (0.021). An F_ST_ value of 0.006 was identified between the Tuva and Mongolian populations. The global F_ST_ value was 0.100. AMOVA computed on the set of 37 samples (excluding the outliers identified in Structure and the Florida Cracker) showed that 10.03% of the variance was accounted for among populations (p = <0.001), 0.53% of the variance was among individuals within populations (p = 0.19), and 89.44% of the variation was within individuals (p<0.001).

## Discussion

These data are gathered from populations that represent tremendous diversity in phenotype and breed specialization. With breeds sampled across four continents, the resulting relationships observed largely reflect similarities of geographic origin, documented breed histories, and shared phenotypes. In general the highest within breed diversity was observed in breeds that are recently derived, continue to allow introgression of other populations, those that have a large census population size, and landrace populations that experience a lesser degree of controlled breeding. Not surprisingly, low diversity is observed in breeds with small census size, relatively old breeds with closed populations, and those with documented founder effects, whether due to population bottlenecks or selective breeding.

A total of seven individuals were identified by parsimony and cluster analysis as outliers with respect to the breed to which they were assigned. The pedigrees of these individuals were unknown. Because it is possible these horses were unknowingly crossbred or subject to mishandling in the field or laboratory, they were excluded from the within-breed analyses to avoid potential bias in indices of diversity. In addition, the potential impact of SNP ascertainment bias on diversity calculations must be acknowledged. The reference genome is from a Thoroughbred mare [40] and SNP identification was based upon the reference genome and data from seven other horses representing six breeds. Therefore, SNPs are generally derived to identify modern variation within the Thoroughbred as well as between the Thoroughbred and these other breeds. Thus, the SNPs identified may reflect an upward bias in diversity indices in the Thoroughbred and closely related breeds [41]. It seems that ascertainment bias may have particularly influenced the results when considering the data sets that have an increased number of loci resulting from relaxed LD pruning. These results show an increase in the relative diversity of the Thoroughbred, breeds with which the Thoroughbred continues to actively interbreed, and the other SNP discovery breeds, with respect to other breeds in the study. This is opposite of what may be expected given the high levels of genome-wide LD in the Thoroughbred. Without considering SNP ascertainment bias, it is expected that measured diversity would increase in breeds with low LD more quickly than in those with high LD, due to greater independence of markers in the former breeds. These SNPs, derived largely from the Thoroughbred, are apparently detecting a higher proportion of Thoroughbred-specific, rare variants and it appears that as more loci are included, more of these Thoroughbred-based variants are assayed, resulting in the observed increase in variation in the Thoroughbred, Thoroughbred-influenced breeds, and breeds used in SNP ascertainment.

The majority of the analyses were performed using 10,536 SNP markers pruned across breeds for LD of r^2^>0.2 as well as MAF of 0.05 or above. Even though additional markers could have been used for analysis, many population-level statistics assume independence of loci. The stringent pruning for LD was therefore undertaken to eliminate bias in the test statistics that may result from substantial breed-specific differences in LD [40], [41]. A truncated data set also helped to make calculations, especially cluster analysis, computationally feasible. On the other hand, diversity indices were calculated after pruning the full data set to r^2^ = 0.2 and r^2^ = 0.4 (using pairwise correlation), and one replicate setting the threshold to R^2^<0.1 (using the variance inflation factor), to examine the effect of allowing for varying levels of LD and therefore varying numbers of loci (see methods).

### Within-breed Diversity

Even considering SNP ascertainment, low diversity as measured by H_e_ was observed in the Thoroughbred as well as the Standardbred, which both experience high selective pressures and are closed populations. Low diversity was also observed in breeds that have undergone a severe population bottleneck, such as the Exmoor and Clydesdale, and breeds that have small census population sizes, such as the Florida Cracker. Although the Thoroughbred is a large population that is widely distributed on a geographic scale, historic records suggest that one sire is responsible for 95% of the paternal lineages in the breed and as few as 30 females make up 94% of maternal lineages [28]. In addition, the population has been largely closed to outside gene flow since the formation of the first stud book in 1791 [42] and individuals within the breed are subject to selective pressure for racing success; therefore low, within-breed diversity is not at all surprising.

Using LD-based calculations, the estimated N_e_ for the Thoroughbred was similar to that found in a UK sample [43] and among the lowest of the study set despite the large census population size and geographic distribution of this breed. Individual inbreeding values based upon observed vs. expected homozygosity indicate that individual Thoroughbred horses show signs of inbreeding, with a mean loss of heterozygosity of 16.3%. This value is slightly larger than that found in [28] (13.9%). Using the same SNP array, [44] also showed inbreeding in the Thoroughbred, and specifically an increase in inbreeding over time. The only breeds with higher f values were the Exmoor, Clydesdale, Mangalarga Paulista, and Shire. Despite low individual diversity, F_IS_ values do not show significant inbreeding in either of the Thoroughbred populations as a whole, or in the Norwegian Standardbred although F_IS_ is significant in the US Standardbred population (discussed below).

The Clydesdale and Exmoor, in addition to having high individual estimated coefficients of inbreeding, also show the lowest within-breed diversity observed in the dataset. A lack of diversity in the Clydesdale and another British draft breed, the Shire, is likely a result of a severe population bottleneck observed in most draft breeds with the onset of industrialization and after the conclusion of World War II (WWII) as well as selection for size and color [45], [46]. The Exmoor pony, considered to be one of the purest native breeds of Britain, has been naturally selected for survival in harsh winter conditions on the moors in southwest England [45], [47]. Similar to the draft breeds, the Exmoor population decreased significantly after WWII to approximately 50 individuals, undoubtedly influencing the diversity observed in this study. The effect of low population size and selection is also reflected in extremely high individual estimates of f within some individuals. Finally, the Mangalarga Paulista shows low levels of heterozygosity, and as discussed below, the greatest divergence as measured by pairwise F_ST_ of all breeds in the study. While these results could be due to geographic distance between this and other breeds, and/or genetic drift, unfortunately these horses were all sampled from only two farms and likely do not represent the entirety of the diversity present in the breed; therefore we cannot rule out sampling error which would inflate the estimated level of divergence between these individuals and the other breeds and result in a decrease in H_e_. However, a lack of diversity in sampling of the breed would not have an effect on estimates of individual inbreeding coefficients, which were among the highest of the entire data set.

Converse to the above examples, high levels of diversity as measured by both H_e_ and N_e_, accompanied by low estimates of inbreeding (f and F_IS_), are observed in the Mongolian, Tuva, and New Forest Pony. The Mongolian and Tuva are unique in that they represent landrace populations that are less managed than the popular breeds of Western Europe and North America; they occupy a diverse range of habitat, have been selected for meat and milk in addition to use in transportation, and originate in the region where domestication was likely to have occurred. The population of Mongolian horses is large and individuals are phenotypically diverse [48]. In 1985, approximately two million Mongolian horses of four different types were estimated to live within the country [45]. The Tuva is not as numerous as the Mongolian but is similar in its purpose and also has high within-breed phenotypic diversity. In addition, it is suggested that the Tuva has experienced outcrossing in order to increase its size and stamina [45] as may also be the case in the Mongolian [49]. Similarly, the New Forest Pony was historically a free-ranging population in Great Britain, but was crossbred until the 1930’s. These traits: old populations, large population size, outcrossing, high phenotypic diversity, and lesser artificial selection/management, result in the high levels of genetic diversity observed. This extent of diversity appears to diminish as populations are restricted by selective pressures into formal breeds.

Other population characteristics are likely the cause of the diversity observed in the Finnhorse, Icelandic, and Miniature. In the case of the Icelandic, the high level of diversity was possibly maintained by a large census population size despite isolation for almost a thousand years and several population bottlenecks due to natural disasters [45]. In the case of the Finnhorse, diversity may be due to within-breed substructure into four sections of the studbook established in 1970: the work horse (draft), trotters, riding horse, and pony [50]. Finally, high diversity in breeds such as the Miniature is likely a result of a diverse founding stock [45], [51], [52]; horses of small size from a variety of geographic regions and bloodlines were utilized in founding the breed, which is defined by phenotype.

All of these three factors, large population size, phenotypic diversity within the breed, and a diversity of founding stock, also lead to the relatively high levels of diversity observed in the Paint and Quarter Horse; in addition, these breeds both allow continued outcrossing between themselves and with the Thoroughbred and have experienced a tremendous population expansion since the formal foundation of the breeds within the past 45–75 years. Due to the relative infancy of these populations, it could be argued that the Paint, Quarter Horse, and other, newly-derived breeds, have not yet had time to undergo the evolutionary processes necessary to be genetically distinct populations as is observed in breeds with longer histories and closed studbooks. However, even with high within-breed diversity and large census population sizes (over 1 million and 4 million worldwide for the Paint and Quarter Horse, respectively), N_e_ for these breeds account for only a fraction of the census size, demonstrating non-random mating and selection. Outcrossing is also continued in the Swiss Warmblood and Hanoverian breeds, which show similar trends in diversity measures as the Quarter Horse and Paint. The relatively low N_e_ in these breeds, accompanied by moderate H_e_ may partially be due to significant crossing with the Thoroughbred, which would contribute long blocks of LD [40], [41], resulting in decreased estimates of N_e_.

Of note in breeds such as the Quarter Horse, Lusitano, and Andalusian, is that despite moderate to high relative levels of H_e_, and low to moderate estimates of f, F_IS_ values in each breed are significantly positive. Significant F_IS_ was also previously observed in the Iberian breeds using microsatellite markers [53]. While selection and inbreeding may be responsible for significant values of F_IS_ in some of these breeds, another instance in which F_IS_ may be significantly positive is in the presence of subpopulation structure within the sample. Evidence of this in the Lusitano and Andalusian is present in parsimony analysis where individuals of the two breeds fall into one clade, but within that clade are two highly supported branches represented by a subset of each breed. In addition, when forcing high values of K in Structure, such as observed at K  = 35, Andalusian and Lusitano individuals fall into one of two clusters with q-value >0.5 in a nonbreed-specific manner (data not shown). These results support [54], which showed potential subpopulation structure in the Lusitano via microsatellite analysis. In the Quarter Horse, subpopulation structure is evident through the evaluation of bloodlines and the selection of popular sires for diverse performance classes. This population substructure in the Quarter Horse has also been demonstrated by marked differences in allele frequencies among performance types (cutting, western pleasure, halter, racing, etc.) [55]. A similar instance is found in the US population of the Standardbred, which also has significant excess homozygosity (F_IS_). Unlike Standardbreds in Europe, which are raced at a trot, those in the US are divergently selected for racing at either the pace or the trot, creating structure within the breed [56].

Finally, several rare populations are included in this dataset. The Caspian is one of the oldest breeds in the Middle East and was thought to be extinct until its recent rediscovery in 1965. The Florida Cracker, a now rare breed, was developed in the United States from feral stock of Iberian descent [57]. The sample size of the Florida Cracker limits the conclusions that can be drawn regarding within-breed diversity. However, the Caspian shows high N_e_, H_e_, and estimates of f, given its rarity. After rediscovery of the breed, which historically was believed to represent a type of landrace population, [58] describes a three-year survey, which found approximately 50 individuals remaining, noting that many could not be considered “pure.” In addition, [33] were unable to show evidence of a recent bottleneck in the Caspian breed. The diversity observed in what are now considered Caspian horses likely stems from high levels of diversity within those individuals that founded the modern population.

### Among-breed Diversity

The expectation of homogeneity within breeds due to closed populations and selection is supported by the results of AMOVA, which show significant variation present among populations, but a non-significant proportion of variance within. However, the variation among samples lends information about current and historic relationships. Observed trends include patterning based upon geographic origin and/or phenotypic similarities, and relatively low divergence observed in comparisons that include breeds with high within-breed diversity. In Structure analysis, K  = 29 was chosen as the most likely value of K; however no single value stood out as the “best” number of clusters to describe these data. Regardless, patterns observed in clustering were also supported by pairwise F_ST_ values, parsimony, PCA, and NJ dendograms.

### High Diversity and Low Divergence – Landrace Breeds

The Mongolian and Tuva populations are believed to have been influential in the spread of horses across Asia and Europe [45], [59]; these landrace populations, harboring high levels of within-breed diversity, were found to be similar to one another, with a pairwise F_ST_ value of 0.006. In addition, with the exception of six Tuva individuals that fell into a clade with the Caspians, both parsimony and NJ analyses place the Tuva and Mongolian into the same clade of each tree. Examining all comparisons, low F_ST_ values were observed between the Mongolian and Tuva compared to the other breeds in this study, supporting the potential role of Eurasian horses of similar type in founding modern stocks. This also aligns with high microsatellite diversity observed in Eastern Eurasian “non-breed” (landrace) populations in [19]. On the other hand, breeds with high diversity in general show lower levels of divergence as measured by F_ST_, while those with low diversity show higher values of F_ST_. Low divergence in breeds with high diversity is expected as variation within a breed may indicate outcrossing with other populations, and high variation also makes these breeds more likely to share variation with others by chance. In contrast, if a breed has little within-breed variation, it is less likely to share genetic variation with another breed by chance, especially with another breed that is relatively homogeneous itself. As demonstrated in human literature, source populations are expected to contain greater diversity than those populations which they found [60], [61]; this is also suggested in the horse by [11], which showed greater mtDNA diversity in Iberian breeds than the recently founded American breeds. If the argument is made that the low F_ST_ values of Tuvas and Mongolians supports their role in founding modern breeds, the same argument could be made for the Quarter Horse, which also shows low levels of pairwise divergence; however that argument would be unreasonable as the Quarter Horse was developed in only the past century. Therefore, the relative values of F_ST_ are informative, but these F_ST_ values and data, which represent modern breeds generally derived from limited founding stock, and subjected to intense artificial selective pressures, cannot be used independently to elucidate the evolution of the modern horse. Also, as is the case for other analyses, while the relationships observed can shed light on the history of breeds, they cannot distinguish between recent admixture and shared ancestry.

### Thoroughbred-influenced Breeds

The Thoroughbred is believed to have founding sires of Arabian, Turk, and Barb ancestry [42], and [28] found that two sires, noted as being Arabian (Godolphin Arabian and Darley Arabian) together contributed to over 20% of the modern population. However, it is likely that the “Arabian” foundation stallions were not Arabians as the breed is known today. It is noted in [62] that the Godolphin Arabian was a Turkoman stallion with partial Arabian blood, while in other work it is suggested he was a Barb [45]. Regardless of the true ancestry of these stallions, restrictions placed upon the export of purebred Arabians during the 16^th^ and 17^th^ centuries, as well as the general use of the term “Arab” for horses of Middle Eastern descent, it is likely other “Arabian” horses with influence on the Thoroughbred breed also had Turkoman, and Barb bloodlines [45], [62]. The pairwise F_ST_ values between the Thoroughbred and the Arabian do not suggest any less divergence than observed between the Thoroughbred and a majority of the other breeds. In addition, at K = 29 the Arabian assigns to the Thoroughbred cluster at only 2.3%. If the Arabian did have significant influence on the Thoroughbred breed, there are several possible explanations for why the supposed Arabian influence is not more apparent. The first is related to SNP ascertainment and the bias of SNPs toward modern variation in Thoroughbred. It is possible that the genes derived from the Arabian are at or near fixation in the Thoroughbred, which would reduce the chance that these SNPs, and the variation described within them are present in the dataset. Another possibility is that the current Arabian sample, taken from the United States, may not reflect the Arabian lineage(s) that were influential in the founding of the Thoroughbred. Finally, as noted above and also suggested elsewhere [63], it may simply be that Arabian bloodlines were not as instrumental in the Thoroughbred breed as once thought or that the initial Arabian influence (and genes) have been selected against or lost to drift during the development of the modern Thoroughbred racehorse.

Within the Thoroughbred itself, divergence between the US and European samples had a significant F_ST_ of 0.004, similar to that observed between the Hanoverian and Swiss Warmblood (0.008) and Mongolian and Tuva (0.006), but larger than the minimally observed value seen between the Paint and the Quarter Horse (0.002). Although artificial insemination is prohibited in the Thoroughbred and would be anticipated to limit gene flow to some extent, the founder effect in the original European Thoroughbred by few high-impact sires and dams, accompanied by shared selective pressures, relatively recent importation of the breed to the United States, and ongoing shipment of horses between continents are likely contributing to the lack of geographic population structure identified by parsimony and cluster analyses.

While within-breed diversity of the Thoroughbred was relatively low, and a notable relationship with the Arabian was not observed, among-breed analysis shows a clear influence of the Thoroughbred on many other breeds. Placed with the Thoroughbred in parsimony analysis are the Hanoverian, Maremmano, and Swiss Warmblood. The Maremmano, an Italian breed, shows a q-value of assignment to the Thoroughbred clade of 0.26 at K  = 29. This is not surprising given reports that Thoroughbreds contributed over 13% of the maternal lineages to the stallion lines within the stud book [64]. Low differentiation of the Maremmano compared to the Hanoverian was also reported in [29], which is logical given similar influence of the Thoroughbred on the Hanoverian. This and the allowed crossing of Thoroughbreds into the Swiss Warmblood population is reflected in minimal measures of divergence between these samples. The continued influence of the Thoroughbred on the Paint and Quarter Horse is also reflected both by low F_ST_ values as well as greater than 30% assignment of the Paint and Quarter Horse to the Thoroughbred cluster. As each of these breeds experience continued gene flow from the Thoroughbred, and had Thoroughbred founding stock, these results are not unexpected. While outcrossing of these breeds is allowed (with restrictions), it is likely that even in cases of breeds with closed stud books, some outcrossing, intentional, unintentional, and/or undocumented, has occurred; it has been demonstrated that historical pedigrees, while helpful, are not always accurate [65]. Issues of outcrossing and individual identification can now be more easily addressed using genetic testing and have the potential to assist managers in decisions regarding breeding and registration.

Finally, the Standardbred samples, which also represent two continents, had a significant pairwise F_ST_ value of 0.020, five-fold greater than that observed between the two Thoroughbred samples. This comparison may reflect geographic structure, the influence of French Trotter bloodlines in the European sample, and selection for pacing horses in the US population. Within the horses included in this study, eight were noted to be pacers. These pacers all fall within one clade of the parsimony tree, supported by a bootstrap value of 98%. The limited sample size does not allow a thorough comparison of the pacing vs. trotting Standardbreds, however significant genetic differentiation between horses of the two racing groups has been reported [56].

### Middle Eastern and Iberian Breeds

The Middle Eastern breeds, Arabian, Akhal Teke, and Caspian, were placed into a single clade of the NJ tree, with the Arabian and Akhal Teke in their own, highly supported clade. This relationship was supported by low values of K in Structure, which placed the Iberian and Middle Eastern breeds into the same cluster until K  = 12. However, parsimony analysis did not support this relationship between the Middle Eastern and Iberian breeds as the Arabian and Akhal Teke individuals were placed into one clade, apart from the Iberian samples and from the Caspians.

In Europe, the Iberian breeds (Lusitano and Andalusian) have only recently been distinguished from one another depending upon the region in which they are bred and divergent selective pressures. In the US, horses of each breed are occasionally interbred [51]. The close relationship between the Andalusian and Lusitano samples in this study is reflected in the minimum F_ST_ value observed in either breed, 0.021. The parsimony tree and PCA also shows that individuals cannot necessarily be distinguished from one another regardless of whether they were sampled in the US or Portugal. Of the two clades that appear to suggest population substructure, one includes only Portuguese Lusitanos while the other includes only US samples, although two are of Portuguese ancestry.

### Iberian and Gaited Breeds

A horse that is considered to be “gaited” naturally moves in a means other than the traditional walk, trot, canter, and gallop. Alternative gaits in horses are distinguished from traditional gaits by their unique footfall pattern and/or rhythm. The genetic basis of gait has recently been investigated and suggests that all modern gaited breeds share a common ancestor as supported by a shared, extended haplotype spanning a variant significantly associated with the ability to pace [66]; [67]. There is a great deal of historical evidence that the shared ancestry of gaited breeds traces back to Iberian bloodlines, in particular to the Spanish Jenette [45], [51]. Influence of the Iberian breeds on modern gaited breeds is seen in early clustering in Structure analysis as well as in the NJ tree where the Puerto Rican Paso Fino and Peruvian Paso are placed on the same branch as the Andalusian and Lusitanos. In addition to Iberian lines, the Narragansett Pacer is often named as instrumental in the founding of American breeds that may gait including the Saddlebred, Standardbred, and Tennessee Walking Horse [45]. Within those breeds, the Tennessee Walking Horse was documented to be greatly influenced by the Saddlebred, Standardbred, and Morgan [68], [69]; and the Standardbred itself had influence from the Thoroughbred and Morgan (among others) [70]. While we do not have samples of the now extinct Narragansett Pacer, our data set does support a close relationship between the Tennessee Walking Horse and Saddlebred, as observed in the NJ and parsimony analysis as well as with the Morgan and Standardbred. At low values of K in cluster analysis, the Tennessee Walking Horse and Saddlebred cluster strongly. In NJ analysis, the Florida Cracker, which has many individuals that demonstrate the ability to gait, is found intermediate to the Iberian and the modern US gaited breeds. Interestingly, the Icelandic, a four- or five-gaited breed, does not show any significant affinity to the other gaited breeds although they share the recently identified major locus that appears to be essential to the ability to perform alternate gaits [66]; [67]. It thus seems that the genetic variant associated with the gait phenotype arose well before the separation of breeds. Instead of clustering with the other gaited breeds, the Icelandic clusters with the Shetland through K = 16 and also is within a highly supported branch of the NJ tree with the Shetland and Miniature. Finally, the influence of the Shetland on the development of the Miniature is observed at all values of K as well as in the parsimony tree where both breeds occupy the same clade.

### Drafts

The Shire and Clydesdale populations share assignment to the same cluster throughout Structure runs. The similarity between these breeds is also seen in a lack of monophyly in the parsimony tree, the sharing of a branch of the NJ tree, positioning in PCA, and a pairwise F_ST_ value of 0.037. The Fell Pony, a British breed, falls out as sister taxa to the British draft horses, the Shire and Clydesdales. However F_ST_ values show that divergence between the Fell Pony and either the Clydesdale or Shire is not significantly less than seen with most other populations. The other branch of the “draft” clade of the NJ tree contains the breeds from the European mainland, the Belgian, Percheron, and Franches-Montagnes; each of these breeds shows monophyly in parsimony analysis. In addition, similarities among draft and light draft breeds are reflected in cluster analyses at K  = 6, which show the populations from the European mainland and Scandinavian Peninsula (Belgian, Finnhorse, Franches-Montagnes, North Swedish Horse, Norwegian Fjord, and Percheron) assign to one cluster with q >0.5. The grouping of the Scandinavian breeds is similar to that previously reported [31]. Geographic relationships are also suggested by the two British breeds, the New Forest Pony and Exmoor that fall just basal to the draft clade in the NJ tree.

### Summary

This data set resulting from a large international collaboration represents the first study in the horse to provide an extensive overview of nuclear genetic diversity within, and relationships among a diverse sample of breeds and landrace populations. These data are now available for use in subsequent studies of population-level relationships and provide a baseline for monitoring changes in breed diversity. With high mtDNA diversity but limited paternal input during domestication, this increased understanding of nuclear diversity within the horse will allow for the identification of genomic regions of importance to breed derivation and will be instrumental in guiding across-breed gene discovery projects.

## Methods

### Ethics Statement

DNA sampling was limited to the collection of blood by jugular venipuncture performed by a licensed veterinarian or from hairs pulled from the mane or tail by the horse owner or researcher. All animal work was conducted in accordance with and approval from the international and national governing bodies at the institutions in which samples were collected (the University of Minnesota Institutional Animal Care and Use Committee (IACUC); the University of Kentucky IACUC; the University College Dublin, Animal Research Ethics Committee; Swiss Law on Animal Protection and Welfare; the Ethical Board of the University of Helsinki; the Animal Health Trust Clinical Research Ethics Committee; Norwegian Animal Research Authority; UK Home Office License; and the Lower Saxon state veterinary office).

### Samples and Genotyping

Tissue samples and previously collected genotypes from 1,060 horses were obtained from members of the EGDC or were obtained by our laboratory. 814 samples representing the 38 populations included in this study were selected from the EDGC sample collection with the goal of obtaining as random of a sample as possible and to minimize close relationships among individuals. In some cases, genotypes were available from breeds collected for genome-wide association studies (GWAS). In all cases, when pedigree information was available, no relationships were allowed at or more recent to the grandsire/dam level. If no pedigrees were available, once genotyping was performed, individuals were removed from the analyses to reduce genome sharing as measured by autosomal estimates of identity by descent (pi hat) values in PLINK [71] greater than 0.3 (after pruning for MAF>0.05). In samples that were obtained as a result of GWAS, “control” individuals were preferentially chosen for inclusion in these analyses. When necessary, DNA isolation from hair roots took place using a modification of the Puregene (Qiagen) protocol for DNA purification from tissue. Modifications include the addition of 750 µl of isopropanol rather than 300, increasing the precipitation spin time to 15 m at 4°C, and washing the pellet twice. Approximately 1 µg of DNA was used for SNP genotyping using the Illumina SNP50 Beadchip according to the manufacturer’s protocol. All genotype calls were extracted from the raw intensity data using GenomeStudio (Illumina) with the minimum gencall score threshold of 0.15. The raw intensity scores were available for all populations with the exception of the Lusitano and Maremmano.

### Data Pruning

SNP discovery was conducted using horses from seven breeds (Akhal Teke, Andalusian, Arabian, Icelandic, Quarter Horse, Standardbred, Thoroughbred) as well as the reference genome of a Thoroughbred mare [41]. To eliminate ascertainment bias as much as possible, horses from the discovery breeds were removed from the dataset, which was then pruned to exclude SNPs with MAF less than 0.05. All horses were then replaced and those SNPs removed from all analyses. In this new, complete data set, SNP markers that failed to genotype in at least 99% of the individuals and SNPs that had a MAF of 0.05 or less across all samples were removed as well as SNPs on ECAX. SNPs that were in LD across breeds were also removed; files used for basic diversity indices were pruned for r^2^<0.1 in PLINK [71] considering 100 SNP windows and moving 25 SNPs per set (–indep-pairwise 100 25 1.11). Allowing for additional LD, data sets were also created for r2<0.2 and 0.4. An additional data set, used for Structure analysis was pruned for R^2^<0.1 in Plink (–indep; R = multiple correlation coefficient), which is similar to the above method but instead of analyzing pairwise relationships of SNPs as in the former method, uses a multiple regression approach upon the SNPs in the analysis window. Files were converted for usage between analyses programs using PLINK, perl script, CONVERT [72] and/or PGDSpider 2.0.1.4 [73].

### Within-breed Diversity

Expected heterozygosity (H_e_) and AMOVA were calculated in Arlequin3.5 [74] on all four data sets. AMOVA was conducted on the primary data set with breeds designated as populations and excluding the Florida Cracker due to small sample size. Analyses were also conducted grouping the two Thoroughbred and Standardbred samples together by breed. F_IS_ was calculated and significance tested on the primary data set, with 10,000 permutations of the data in Genetix [75]. Individual inbreeding coefficients (f) were calculated in PLINK based upon loss of heterozygosity (–het).

### Among Breed Relationships

Pairwise F_ST_ values were calculated on the primary, 10,536 SNP dataset in Arlequin3.5 [74] using Reynolds’ distance [76] with significance tested using 20,000 permutations.

A neighbor joining (NJ) cladogram was built using breed allele frequencies calculated from the primary SNP set using the packages seqboot, gendist, neighbor, and consense and Nei’s genetic distance [38] in PHYLIP ver3.69 [77]. Bootstrap support from 1,000 iterations of the data was used to assess support for the resulting majority rule consensus cladogram.

A parsimony cladogram was constructed using 10,066 SNP markers pruned from the original data set using the MAF and genotyping rate criteria as above and allowing for R^2^<0.2 (–indep-pairwise). The domestic ass was included as an outgroup for traditional and new-technology searches in TNT [78].

Principal component analysis (PCA) was conducted in snpStats in R (http://cran.r-project.org) on the full SNP set consisting of all 814 individuals and 38,755 autosomal SNPs (pruned only for MAF and genotyping rate).

### Cluster Analysis

Clustering of breeds into genetic groups was examined using the program Structure 2.3.3 [79], [80] assuming K  = 1 to 45. The Structure algorithm included the admixture model and correlated allele frequencies. Three iterations of each K value were conducted with 35,000 MCMC repetitions (15,000 burn-in). The convergence of Structure runs was evaluated by equilibrium of alpha and likelihood scores. The value of K most suitable to explain the diversity in these data was predicted by the highest mean estimated ln P(X|K) while minimizing variance and also making biological sense [80], [81]. The replicates from each run of K were input into CLUMPP [82] and the average cluster membership calculated using the LargeK Greedy algorithm. Output from CLUMPP was visualized in Distruct [83].

### Effective Population Size

To estimate effective population size (N_e_), the full set of 54,602 SNP markers was pruned within each population to remove those with MAF <0.01 and genotyping rate of <0.05. Pairwise r^2^ values between remaining SNPs were calculated in Haploview [84], for each population considering intermarker distances from 0 to 4 Mb in 50 kb increments. Values of N_e_ were calculated using the method of [37], which includes a correction for small sample size and the assumption that 1 Mb  = 1 cM.

### Data Access

All SNP genotype data are available at the NAGPR Community Data Repository (animalgenome.org) for the purpose of reconstructing the analyses. The only exception is the data collected from the Tennessee Walking Horse, which, under agreement from the granting agency (to the University of Minnesota from the Foundation for the Advancement of the Tennessee Walking Show Horse (FAST) and the Tennessee Walking Horse Foundation (TWHF)), is only available under a Material Transfer Agreement (MTA) between interested individuals and the University of Minnesota.

## Supporting Information

Figure S1 **Example of LD decay over 2 Mb in 9 breeds of horse.** Decay of linkage disequilibrium over 2Mb for 9 of the 36 breeds. Landrace populations such as the Mongolian, and large and/or diverse breeds such as the Finnhorse and Quarter Horse, show more rapid decay than those with small population sizes and less diversity (e.g. Clydesdale, Tennessee Walking Horse).
(TIFF)

Figure S2 **Parsimony relationship among Lusitano and Andalusian individuals.** Portion of the parsimony clade shown in Figure 1 consisting of the Lusitano (dark blue) and US Andalusian (light blue) individuals. Bootstrap values greater than 50% are shown. Asterisks indicate horses sampled in the US which were noted to be of Portuguese ancestry.
(TIFF)

Figure S3 **Principal component 1 vs. 2 as determined from 814 horses from 38 populations.** Principal components 1 and 2 as determined from 38,755 SNPs (pruned for MAF and genotyping rate). All 814 individuals are included in the plot.
(TIFF)

Figure S4 **Mean of estimated ln P(X|K) for each run in Structure.** Mean of estimated ln P(X|K) for each of the three runs for K = 1−45 in Structure.
(TIFF)

Figure S5 **Bayesian clustering output for additional values of K in 814 horses of 38 populations.** Structure output for additional values of K. Each individual is represented by one vertical line with the proportion of assignment to each cluster shown on the y axis and colored by cluster.
(TIF)

Figure S6 **Parsimony relationship among Thoroughbred horses from the US and UK/Ire.** The branch of the parsimony clade shown in Figure 1 containing the US and UK/Ire Thoroughbreds. Horses sampled in the UK/Ire are noted with an asterisk. Bootstrap values >50% are shown.
(TIF)

Figure S7 **Parsimony relationship among Standardbreds from the US and Norway.** The branch of the parsimony clade shown in Figure 1 containing the US (yellow) and Norwegian (green) Standardbreds. Bootstrap values >50% are shown. The asterisks indicate individuals that are pacing horses.
(TIFF)

Table S1 Cluster to which each population maximally assigns and corresponding q-value for K = 2 to 45. Highest breed q-value of assignment and cluster identity (#) for each value of K examined in Structure. The cluster ID # is not carried through across values of K.
(PDF)

Table S2 **Proportion of assignment for 38 horse populations to each of K = 29 clusters.** Proportion of assignment to each of K = 29 clusters as determined in Structure. The largest proportion of assignment for each population is outlined and shown in bold; those with 30–50% assignment are shown in italic. The top row notes the breed(s) with >50% of assignment to each of the 29 clusters. This analysis was performed without removal of outlier individuals.
(PDF)
