# Supplementary figures and images for: Genetic Diversity in the Modern Horse Illustrated from Genome-Wide SNP Data
Source: PLoS One. 2013 Jan 30;8(1):e54997. doi: 10.1371/journal.pone.0054997 (PMC3559798; doi:10.1371/journal.pone.0054997)

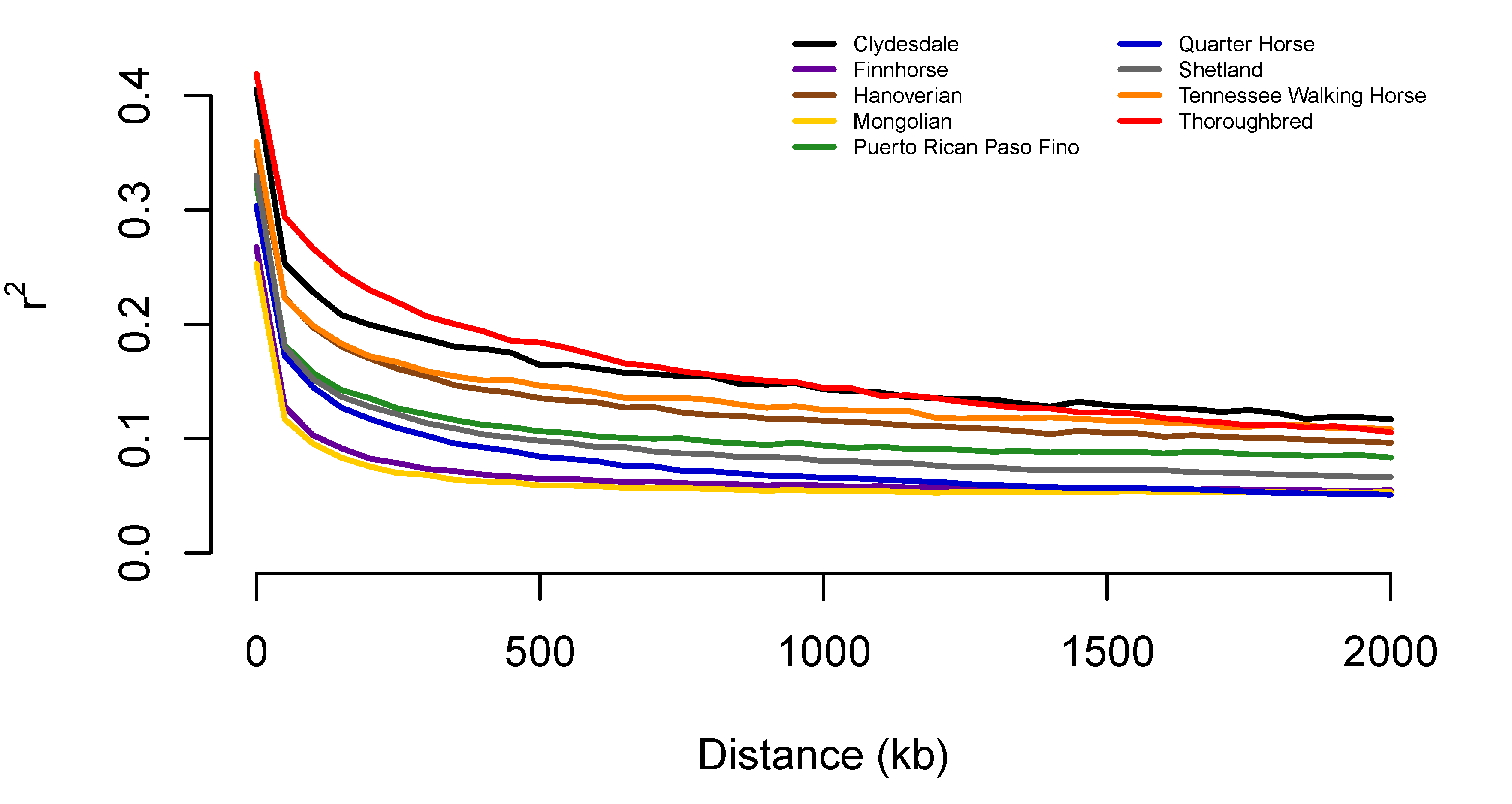

Supplement: Figure S1 — Example of LD decay over 2 Mb in 9 breeds of horse. Decay of linkage disequilibrium over 2Mb for 9 of the 36 breeds. Landrace populations such as the Mongolian, and large and/or diverse breeds such as the Finnhorse and Quarter Horse, show more rapid decay than those with small population sizes and less diversity (e.g. Clydesdale, Tennessee Walking Horse). (TIFF) [file pone.0054997.s001.tiff]

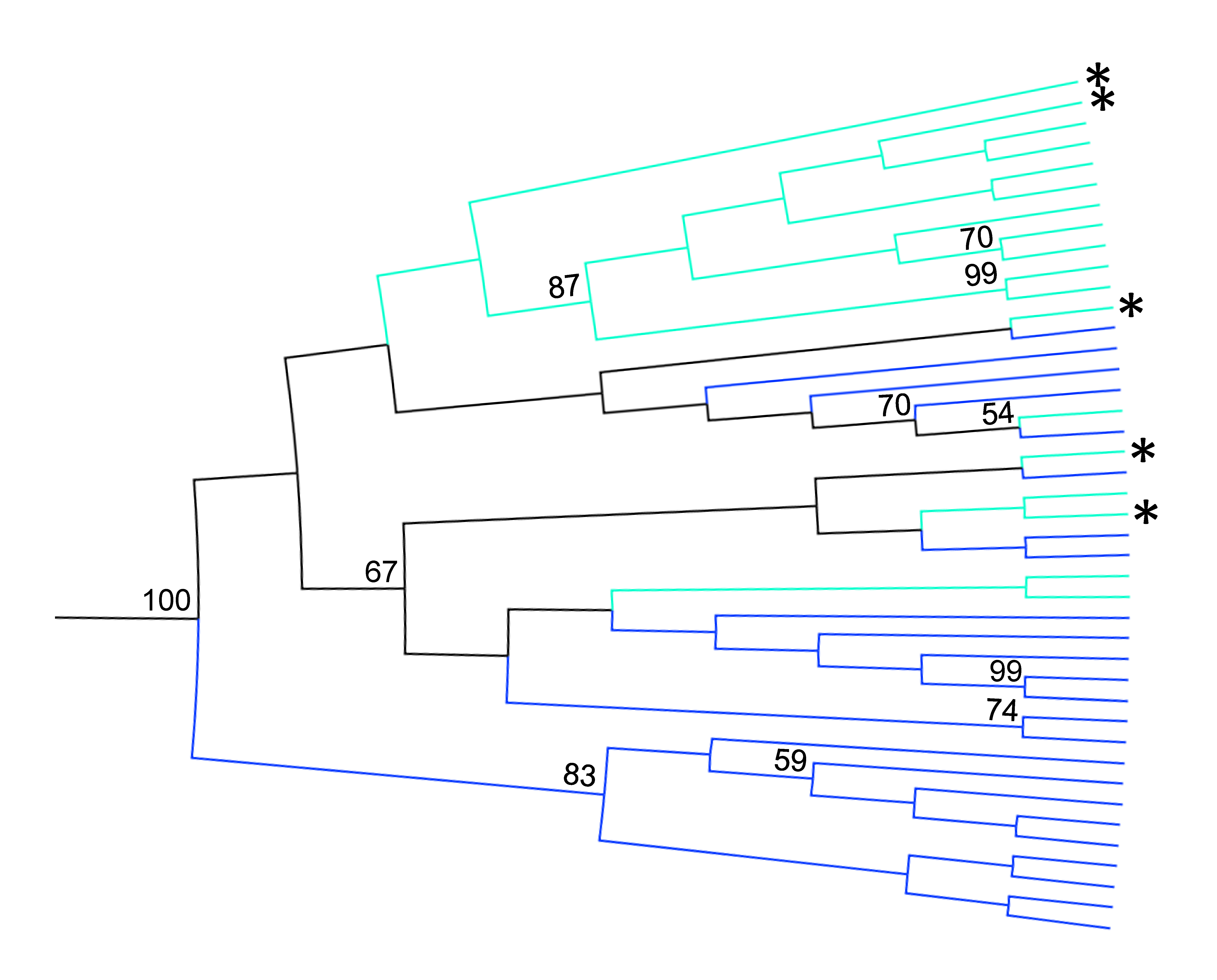

Supplement: Figure S2 — Parsimony relationship among Lusitano and Andalusian individuals. Portion of the parsimony clade shown in Figure 1 consisting of the Lusitano (dark blue) and US Andalusian (light blue) individuals. Bootstrap values greater than 50% are shown. Asterisks indicate horses sampled in the US which were noted to be of Portuguese ancestry. (TIFF) [file pone.0054997.s002.tiff]

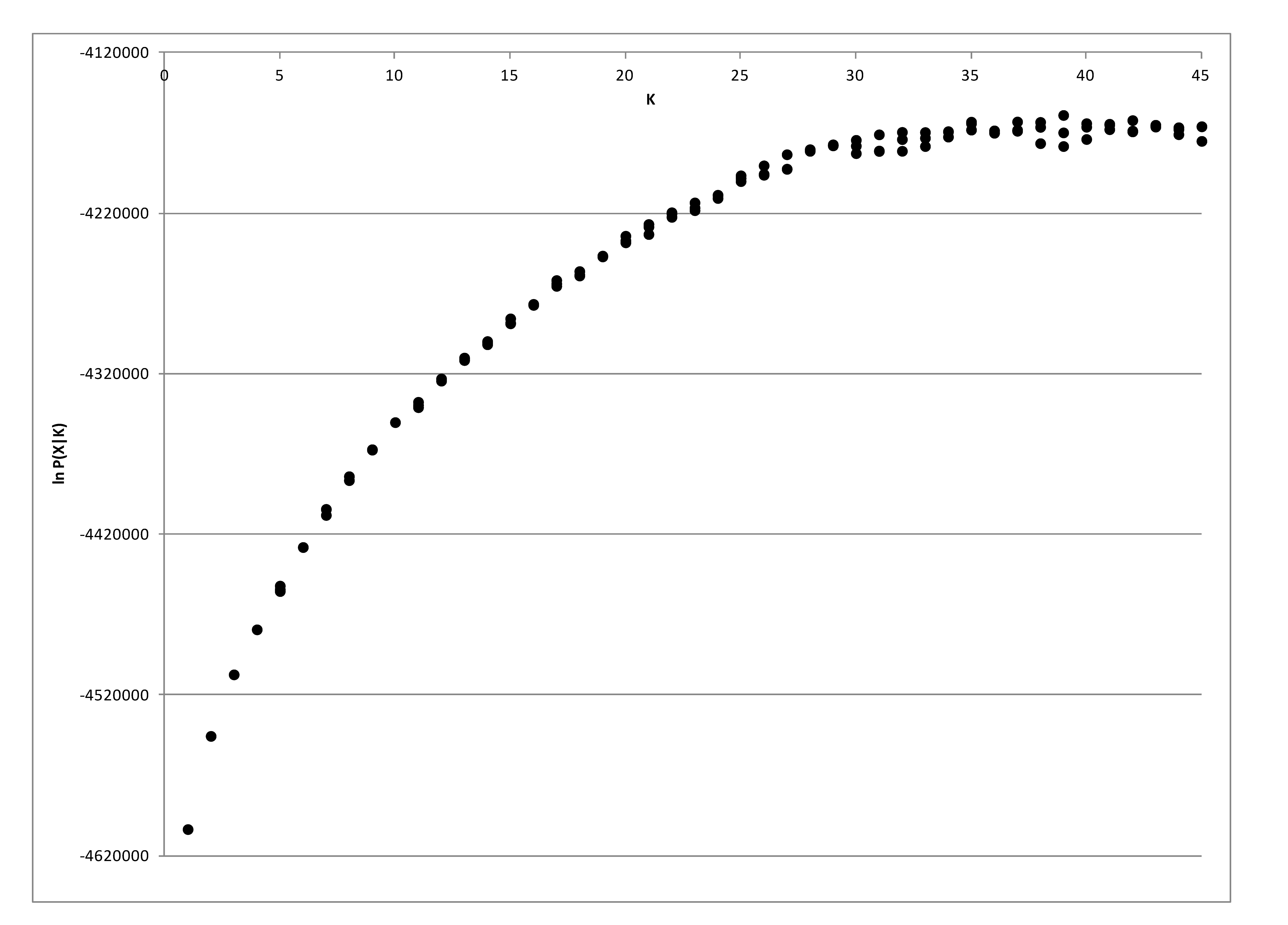

Supplement: Figure S4 — Mean of estimated ln P(X|K) for each run in Structure. Mean of estimated ln P(X|K) for each of the three runs for K = 1−45 in Structure. (TIFF) [file pone.0054997.s004.tiff]

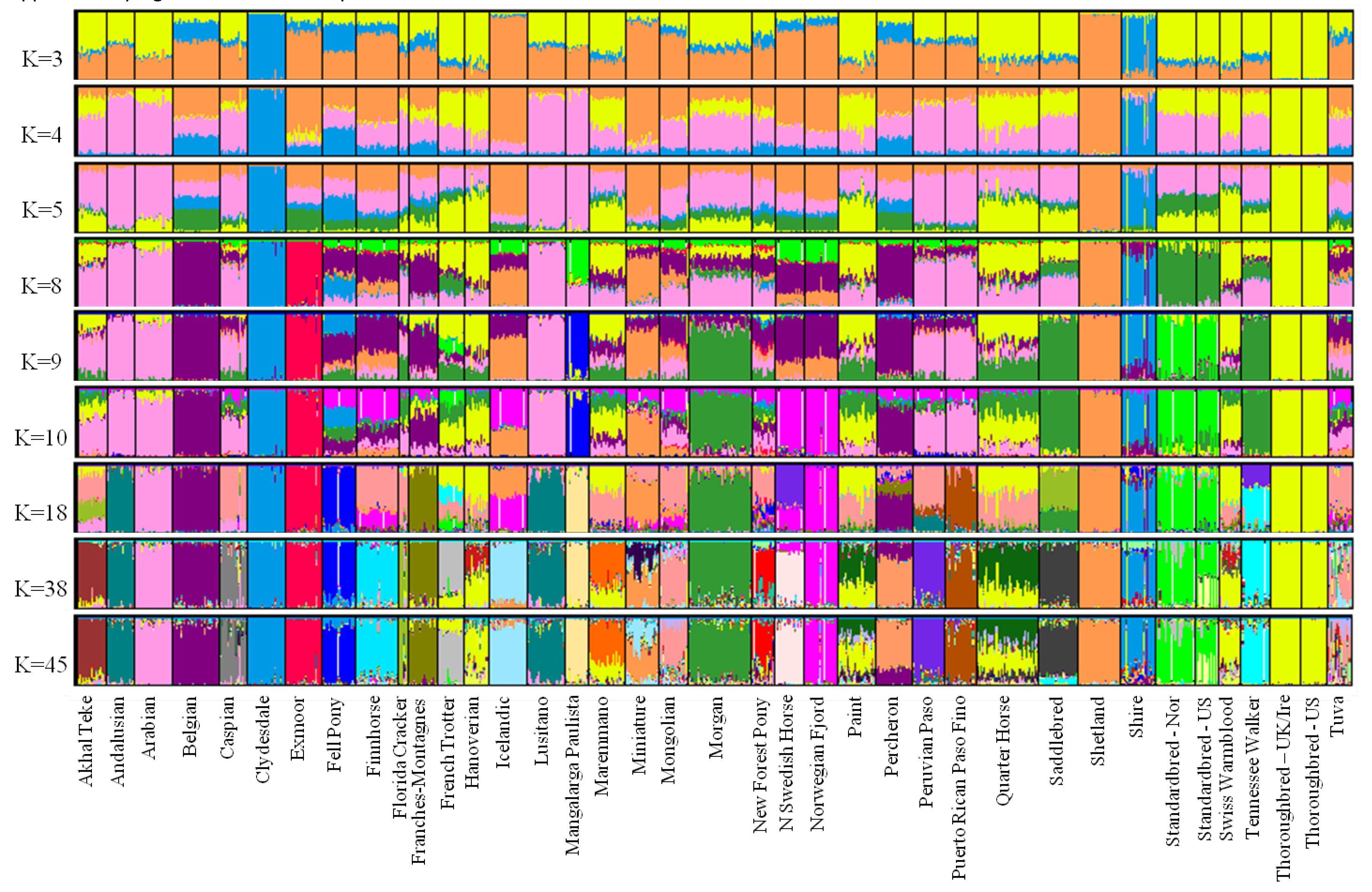

Supplement: Figure S5 — Bayesian clustering output for additional values of K in 814 horses of 38 populations. Structure output for additional values of K. Each individual is represented by one vertical line with the proportion of assignment to each cluster shown on the y axis and colored by cluster. (TIF) [file pone.0054997.s005.tiff]

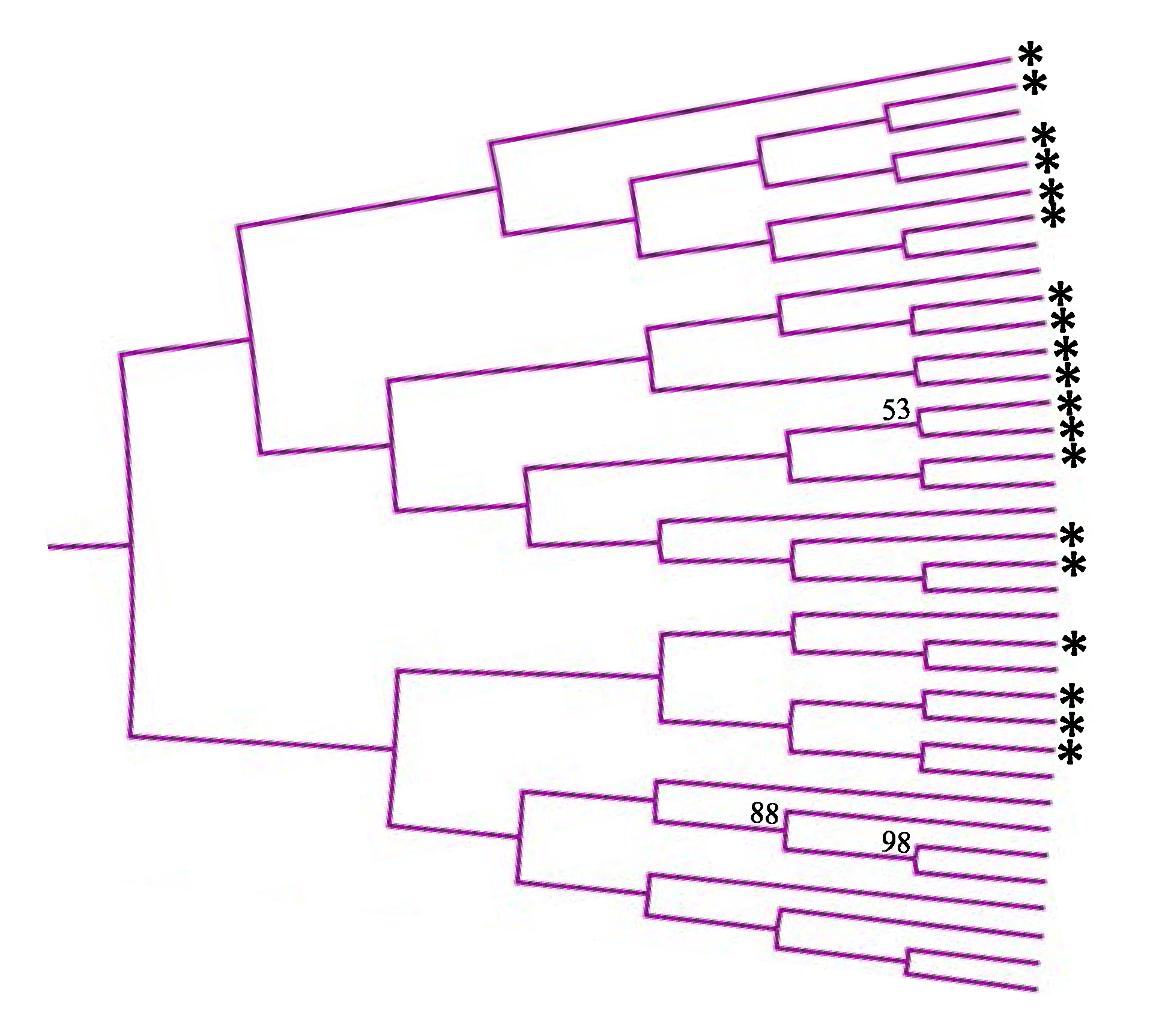

Supplement: Figure S6 — Parsimony relationship among Thoroughbred horses from the US and UK/Ire. The branch of the parsimony clade shown in Figure 1 containing the US and UK/Ire Thoroughbreds. Horses sampled in the UK/Ire are noted with an asterisk. Bootstrap values >50% are shown. (TIF) [file pone.0054997.s006.tiff]

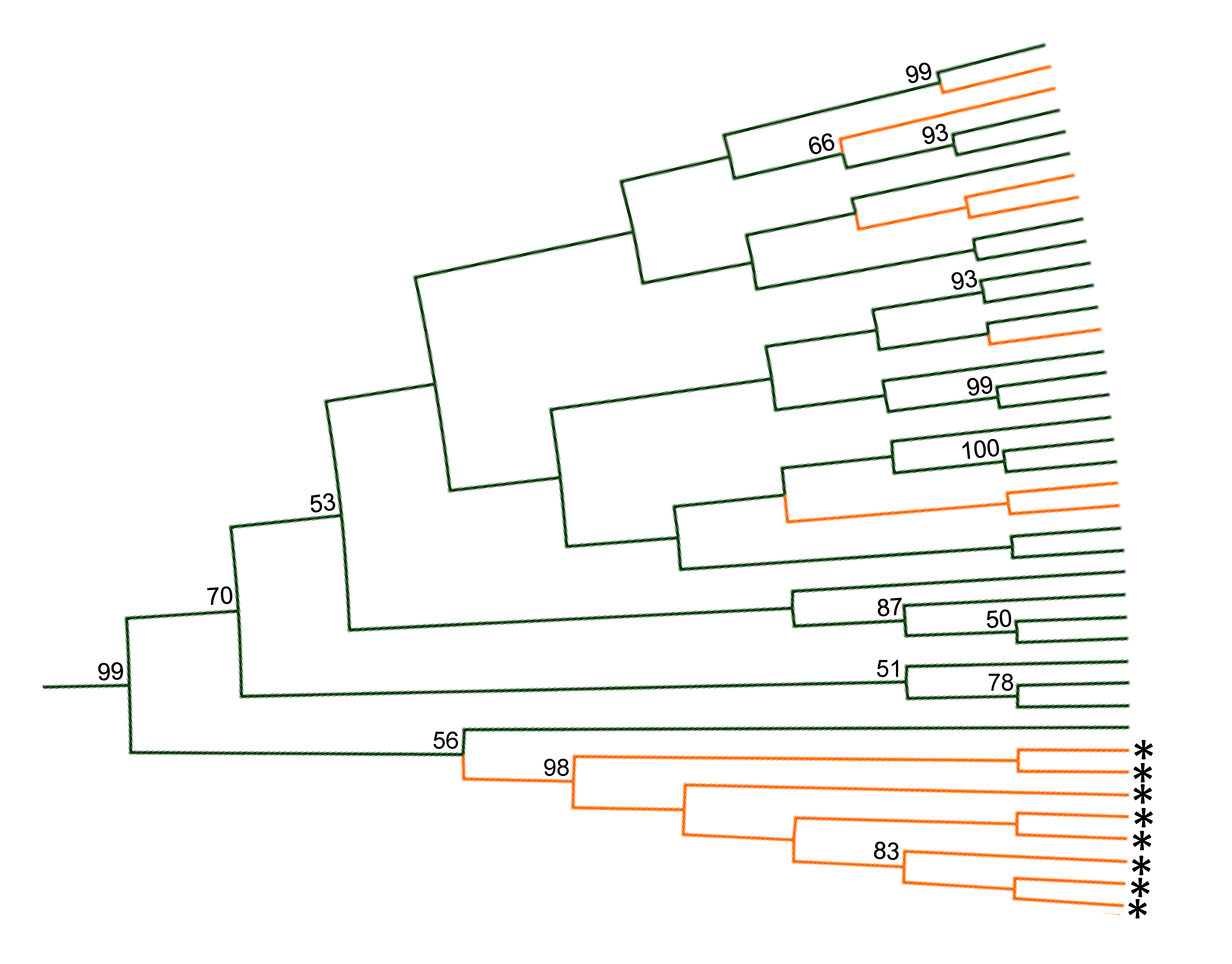

Supplement: Figure S7 — Parsimony relationship among Standardbreds from the US and Norway. The branch of the parsimony clade shown in Figure 1 containing the US (yellow) and Norwegian (green) Standardbreds. Bootstrap values >50% are shown. The asterisks indicate individuals that are pacing horses. (TIFF) [file pone.0054997.s007.tiff]
