# Supplementary material for: Genetic Diversity in the Modern Horse Illustrated from Genome-Wide SNP Data
Source: PLoS One. 2013 Jan 30;8(1):e54997. doi: 10.1371/journal.pone.0054997 (PMC3559798; doi:10.1371/journal.pone.0054997)
Supplement: Table S1 — Cluster to which each population maximally assigns and corresponding q-value for K = 2 to 45. Highest breed q-value of assignment and cluster identity (#) for each value of K examined in Structure. The cluster ID # is not carried through across values of K. (PDF) [file pone.0054997.s008.pdf]

Table S1. Highest breed q-value of assignment and cluster identity (#) for each value of K examined in Structure. The cluster ID # is not carried through across values of K.

K	Akhal Teke	Andalusian	Arabian	Belgian	Caspian	Clydesdale	Exmoor	Fell Pony	Finnhorse	Florida Cracker	Franches-Montagnes	French Trotter	Hanoverian
2	0.58 (1)	0.53 (1)	0.67 (2)	0.86 (1)	0.56 (1)	0.99 (1)	0.84 (1)	0.83 (1)	0.81 (1)	0.51 (2)	0.65 (1)	0.71 (2)	0.76 (2)
3	0.56 (2)	0.52 (2)	0.64 (2)	0.58 (1)	0.50 (1)	0.98 (3)	0.74 (1)	0.42 (1,3)	0.70 (1)	0.49 (2)	0.47 (1)	0.70 (2)	0.75 (2)
4	0.56 (2)	0.87 (2)	0.78 (2)	0.42 (4)	0.62 (2)	0.98 (1)	0.67 (4)	0.41 (1)	0.50 (4)	0.54 (2)	0.32 (2)	0.51 (3)	0.62 (3)
5	0.58 (5)	0.93 (5)	0.78 (5)	0.34 (3)	0.66 (5)	0.98 (4)	0.45 (3)	0.41 (4)	0.46 (3)	0.52 (5)	0.36 (5)	0.41 (2)	0.60 (2)
6	0.56 (6)	0.86 (6)	0.82 (6)	0.97 (1)	0.59 (6)	0.98 (5)	0.94 (4)	0.41 (1)	0.54 (1)	0.52 (6)	0.63 (1)	0.49 (3)	0.61 (3)
7	0.39 (3)	0.69 (3)	0.60 (3)	0.93 (6)	0.42 (3)	0.98 (5)	0.94 (1)	0.48 (6)	0.61 (6)	0.32 (3,6)	0.66 (6)	0.43 (7)	0.62 (7)
8	0.64 (2)	0.94 (2)	0.87 (2)	0.98 (5)	0.63 (2)	0.98 (7)	0.94 (4)	0.41 (5)	0.52 (5)	0.51 (2)	0.62 (5)	0.41 (8)	0.60 (8)
9	0.52 (2)	0.94 (2)	0.84 (2)	0.98 (5)	0.52 (2)	0.98 (7)	0.94 (4)	0.41 (5)	0.54 (5)	0.36 (1)	0.64 (5)	0.36 (8)	0.56 (8)
10	0.52 (4)	0.94 (4)	0.85 (4)	0.97 (5)	0.52 (4)	0.98 (9)	0.94 (3)	0.29 (9)	0.47 (10)	0.38 (7)	0.50 (5)	0.36 (6)	0.56 (6)
11	0.52 (4)	0.94 (4)	0.86 (4)	0.97 (6)	0.53 (4)	0.98 (7)	0.94 (10)	0.29 (7)	0.47 (5)	0.33 (4)	0.50 (6)	0.36 (9)	0.56 (9)
12	0.52 (9)	0.93 (10)	0.96 (9)	0.97 (3)	0.37 (9)	0.98 (12)	0.94 (8)	0.29 (12)	0.49 (5)	0.32 (7)	0.50 (3)	0.35 (2)	0.56 (2)
13	0.54 (8)	0.93 (10)	0.96 (8)	0.96 (9)	0.39 (8)	0.98 (2)	0.94 (12)	0.29 (2)	0.49 (1)	0.33 (4)	0.49 (9)	0.35 (6)	0.55 (6)
14	0.52 (8)	0.93 (11)	0.96 (8)	0.97 (1)	0.38 (8)	0.98 (12)	0.94 (6)	0.29 (12)	0.42 (9)	0.36 (7)	0.52 (1)	0.35 (2)	0.55 (2)
15	0.58 (9)	0.89 (9)	0.90 (9)	0.97 (5)	0.57 (9)	0.97 (7)	0.94 (2)	0.90 (11)	0.36 (13)	0.29 (9)	0.51 (5)	0.36 (8)	0.57 (8)
16	0.58 (16)	0.93 (12)	0.96 (16)	0.96 (5)	0.42 (16)	0.97 (10)	0.94 (6)	0.89 (15)	0.36 (7)	0.21 (9)	0.51 (5)	0.36 (11)	0.57 (11)
17	0.53 (13)	0.93 (7)	0.96 (13)	0.97 (6)	0.38 (13)	0.97 (5)	0.94 (3)	0.90 (15)	0.31 (11)	0.28 (1)	0.51 (6)	0.36 (2)	0.57 (2)
18	0.87 (2)	0.93 (1)	0.94 (6)	0.95 (4)	0.73 (10)	0.98 (16)	0.95 (8)	0.90 (11)	0.72 (10)	0.61 (10)	0.90 (17)	0.37 (18)	0.56 (18)
19	0.87 (19)	0.93 (3)	0.94 (15)	0.95(8)	0.75 (7)	0.98 (6)	0.95 (12)	0.89 (4)	0.60 (7)	0.72 (7)	0.91 (14)	0.35 (5)	0.56 (5)
20	0.86 (10)	0.93 (6)	0.94 (3)	0.95 (2)	0.68 (11)	0.97 (19)	0.94 (14)	0.90 (20)	0.74 (11)	0.73 (15)	0.68 (11)	0.37 (7)	0.57 (7)
21	0.87 (20)	0.93 (12)	0.94 (11)	0.95 (19)	0.56 (2)	0.98 (15)	0.94 (13)	0.89 (1)	0.92 (2)	0.80 (5)	0.91 (4)	0.35 (14)	0.56 (14)
22	0.86 (3)	0.92 (6)	0.93 (4)	0.95 (22)	0.78 (20)	0.98 (14)	0.94 (15)	0.90 (16)	0.91 (13)	0.79 (20)	0.90 (8)	0.35 (17)	0.55 (17)
23	0.88 (9)	0.92 (6)	0.94 (23)	0.94 (10)	0.49 (11)	0.98 (5)	0.94 (4)	0.88 (2)	0.94 (11)	0.73 (19)	0.89 (17)	0.76 (3)	0.51 (8)
24	0.88 (4)	0.92 (24)	0.94 (18)	0.95 (13)	0.83 (22)	0.98 (20)	0.94 (15)	0.88 (8)	0.91 (2)	0.73 (10)	0.91 (11)	0.77 (19)	0.51 (3)
25	0.87 (24)	0.92 (9)	0.94 (16)	0.92 (20)	0.79 (7)	0.98 (11)	0.94 (17)	0.89 (8)	0.92 (18)	0.72 (25)	0.89 (19)	0.76 (2)	0.53 (15)
26	0.87 (24)	0.92 (14)	0.94 (16)	0.92 (21)	0.79 (12)	0.98 (15)	0.94 (17)	0.87 (20)	0.92 (4)	0.72 (26)	0.89 (19)	0.77 (10)	0.51 (25)
27	0.87 (14)	0.91 (21)	0.94 (17)	0.92 (16)	0.78 (4)	0.98 (8)	0.94 (2)	0.89 (5)	0.91 (13)	0.73 (26)	0.90 (23)	0.77 (20)	0.50 (10)
28	0.86 (2)	0.71 (4)	0.93 (9)	0.92 (28)	0.73 (5)	0.98 (27)	0.94 (19)	0.87 (12)	0.85 (7)	0.78 (17)	0.88 (23)	0.76 (8)	0.52 (26)
29	0.87 (2)	0.92 (7)	0.93 (17)	0.92 (21)	0.74 (27)	0.98 (9)	0.94 (3)	0.85 (20)	0.87 (22)	0.72 (26)	0.89 (5)	0.76 (18)	0.51 (13)
30	0.87 (13)	0.92 (4)	0.93 (26)	0.92 (15)	0.74 (2)	0.98 (21)	0.94 (22)	0.87(10)	0.87 (16)	0.72 (5)	0.88 (1)	0.75 (29)	0.52 (30)
31	0.86 (21)	0.91 (3)	0.93 (7)	0.92 (14)	0.74 (29)	0.98 (6)	0.94 (10)	0.84 (9)	0.87 (17)	0.72 (28)	0.88 (5)	0.76 (18)	0.52 (12)
32	0.87 (4)	0.91 (30)	0.93 (25)	0.91 (5)	0.72 (22)	0.97 (13)	0.94 (32)	0.84 (8)	0.84 (15)	0.72 (14)	0.87 (29)	0.75 (10)	0.46 (17)
33	0.86 (22)	0.87 (24)	0.93 (29)	0.91 (11)	0.71 (3)	0.98 (26)	0.94 (2)	0.85 (25)	0.84 (32)	0.72 (17)	0.87 (20)	0.75 (13)	0.48 (27)
34	0.85 (24)	0.91 (8)	0.93 (30)	0.91 (23)	0.72 (2)	0.98 (22)	0.94 (7)	0.84 (29)	0.84 (34)	0.72 (1)	0.86 (3)	0.75 (28)	0.46 (5)
35	0.60 (32)	0.70 (16)	0.93 (14)	0.91 (4)	0.73 (3)	0.98 (10)	0.94 (30)	0.84 (25)	0.84 (20)	0.72 (5)	0.87 (33)	0.75 (6)	0.49 (19)
36	0.86 (5)	0.91 (8)	0.93 (23)	0.91 (4)	0.72 (30)	0.98 (15)	0.94 (22)	0.85 (33)	0.84 (27)	0.72 (25)	0.87 (14)	0.75 (28)	0.47 (18)
37	0.85 (6)	0.70 (17)	0.93 (27)	0.91 (26)	0.73 (9)	0.98 (1)	0.94 (28)	0.85 (19)	0.83 (8)	0.72 (23)	0.87 (30)	0.76 (33)	0.49 (21)
38	0.85 (13)	0.90 (6)	0.93 (2)	0.91 (25)	0.73 (7)	0.96 (28)	0.94 (12)	0.84 (34)	0.83 (24)	0.72 (8)	0.86 (31)	0.75 (33)	0.47 (4)
39	0.87 (26)	0.69 (5)	0.93 (22)	0.91 (24)	0.72 (39)	0.98 (4)	0.94 (8)	0.83 (29)	0.83 (19)	0.72 (10)	0.86 (37)	0.75 (35)	0.48 (3)
40	0.86 (26)	0.88 (37)	0.93 (5)	0.91 (39)	0.71 (14)	0.98 (38)	0.94 (6)	0.84 (35)	0.82 (23)	0.71 (36)	0.86 (2)	0.76 (31)	0.47 (1)
41	0.85 (10)	0.89 (26)	0.93 (1)	0.91 (36)	0.68 (37)	0.97 (24)	0.94 (5)	0.85 (27)	0.81 (18)	0.71 (13)	0.87 (11)	0.74 (14)	0.47 (6)
42	0.85 (5)	0.84 (41)	0.92 (19)	0.91 (32)	0.72 (22)	0.98 (2)	0.94 (42)	0.84 (23)	0.82 (20)	0.72 (29)	0.86 (40)	0.75 (14)	0.46 (24)
43	0.86 (40)	0.88 (34)	0.93 (29)	0.91 (20)	0.72 (14)	0.95 (26)	0.94 (8)	0.84 (31)	0.81 (11)	0.71 (27)	0.86 (23)	0.75 (32)	0.46 (7)
44	0.86 (44)	0.85 (12)	0.93 (40)	0.90 (38)	0.66 (20)	0.98 (17)	0.94 (37)	0.85 (33)	0.81 (8)	0.72 (42)	0.87 (36)	0.75 (30)	0.48 (1)
45	0.85 (38)	0.89 (17)	0.92 (5)	0.91 (2)	0.70 (13)	0.97 (45)	0.94 (34)	0.85 (22)	0.82 (35)	0.72 (40)	0.86 (7)	0.74 (12)	0.50 (26)

Table S1 (continued)

K	Icelandic	Lusitano	Mangalarga Paulista	Maremmano	Miniature	Mongolian	Morgan	New Forest Pony	North Swedish Horse	Norwegian Fjord	Paint	Percheron	Peruvian Paso
2	0.94 (1)	0.52 (1)	0.55 (2)	0.62 (2)	0.87 (1)	0.74 (1)	0.50 (1,2)	0.66 (1)	0.85 (1)	0.89 (1)	0.70 (2)	0.83 (1)	0.57 (1)
3	0.95 (1)	0.49 (1)	0.51 (2)	0.60 (2)	0.87 (1)	0.66 (1)	0.48 (2)	0.50 (1)	0.74 (1)	0.82 (1)	0.69 (2)	0.57 (1)	0.53 (1)
4	0.81 (4)	0.86 (2)	0.85 (2)	0.44 (3)	0.78 (4)	0.43 (4)	0.53 (2)	0.40 (2)	0.54 (4)	0.64 (4)	0.52 (3)	0.38 (4)	0.72 (2)
5	0.80 (3)	0.92 (5)	0.95 (5)	0.43 (2)	0.77 (3)	0.45 (5)	0.50 (5)	0.43 (5)	0.51 (3)	0.62 (3)	0.49 (2)	0.38 (5)	0.76 (5)
6	0.63 (2)	0.87 (6)	0.96 (6)	0.43 (3)	0.69 (2)	0.40 (1)	0.49 (6)	0.35 (1)	0.64 (1)	0.63 (1)	0.50 (3)	0.85 (1)	0.70 (6)
7	0.63 (4)	0.69 (3)	0.95 (3)	0.45 (7)	0.69 (4)	0.48 (6)	0.41 (6)	0.43 (6)	0.68 (6)	0.64 (6)	0.51 (7)	0.85 (6)	0.52 (3)
8	0.64 (1)	0.94 (2)	0.93 (6)	0.42 (8)	0.70 (1)	0.38 (5)	0.49 (2)	0.34 (5)	0.66 (5)	0.63 (5)	0.49 (8)	0.85 (5)	0.70 (2)
9	0.64 (3)	0.93 (2)	0.92 (9)	0.37 (8)	0.70 (3)	0.39 (5)	0.79 (1)	0.33 (5)	0.68 (5)	0.63 (5)	0.42 (8)	0.84 (5)	0.61 (2)
10	0.58 (10)	0.93 (4)	0.92 (1)	0.37 (6)	0.63 (2)	0.34 (10)	0.83 (7)	0.22 (4)	0.92 (10)	0.96 (10)	0.42 (6)	0.71 (5)	0.60 (4)
11	0.58 (5)	0.94 (4)	0.92 (11)	0.38 (9)	0.63 (1)	0.33 (5)	0.88 (2)	0.22 (4)	0.92 (5)	0.96 (5)	0.43 (9)	0.71 (6)	0.59 (4)
12	0.59 (5)	0.84 (10)	0.92 (4)	0.37 (2)	0.63 (1)	0.34 (5)	0.88 (7)	0.20 (5)	0.93 (5)	0.96 (5)	0.42 (2)	0.71 (3)	0.53 (10)
13	0.59 (1)	0.84 (10)	0.92 (7)	0.37 (6)	0.63 (3)	0.36 (1)	0.89 (4)	0.22 (1)	0.93 (1)	0.96 (1)	0.42 (6)	0.70 (9)	0.32 (10)
14	0.62 (9)	0.84 (11)	0.92 (3)	0.37 (2)	0.62 (10)	0.28 (9)	0.89 (7)	0.22 (7)	0.90 (13)	0.95 (9)	0.42 (2)	0.73 (1)	0.32 (11)
15	0.57 (13)	0.90 (9)	0.92 (10)	0.38 (8)	0.64 (15)	0.23 (13)	0.89 (14)	0.22 (9)	0.90 (6)	0.95 (13)	0.44 (8)	0.70 (5)	0.46 (9)
16	0.57 (7)	0.84 (12)	0.92 (3)	0.38 (11)	0.64 (14)	0.23 (7)	0.90 (9)	0.16 (15)	0.90 (1)	0.95 (7)	0.43 (11)	0.69 (5)	0.31 (12)
17	0.96 (11)	0.84 (7)	0.92 (9)	0.38 (2)	0.56 (4)	0.19 (11)	0.92 (1)	0.18 (1)	0.91 (17)	0.93 (8)	0.43 (2)	0.69 (6)	0.31 (7)
18	0.53 (14)	0.83 (1)	0.93 (5)	0.52 (10)	0.63 (9)	0.63 (10)	0.75 (12)	0.51 (10)	0.90 (7)	0.95 (14)	0.43 (18)	0.54 (4)	0.59 (10)
19	0.95 (11)	0.83 (3)	0.92 (16)	0.50 (7)	0.55 (18)	0.59 (7)	0.87 (9)	0.48 (7)	0.92 (2)	0.94 (2)	0.45 (7)	0.53 (8)	0.64 (7)
20	0.95 (13)	0.84 (6)	0.92 (17)	0.48 (11)	0.55 (1)	0.62 (11)	0.87 (16)	0.48 (11)	0.91 (12)	0.93 (5)	0.44 (7)	0.57 (2)	0.52 (11)
21	0.94 (16)	0.83 (12)	0.92 (21)	0.39 (14)	0.56 (9)	0.50 (2)	0.88 (17)	0.28 (2)	0.91 (8)	0.93 (3)	0.47 (5)	0.53 (19)	0.72 (5)
22	0.95 (21)	0.81 (6)	0.92 (5)	0.38 (17)	0.57 (18)	0.30 (20)	0.86 (19)	0.25 (20)	0.91 (12)	0.93 (7)	0.46 (20)	0.54 (22)	0.90 (10)
23	0.90 (20)	0.82 (6)	0.92 (22)	0.38 (8)	0.57 (1)	0.52 (11)	0.87 (21)	0.33 (11)	0.89 (12)	0.94 (12)	0.55 (13)	0.53 (10)	0.90 (18)
24	0.94 (5)	0.82 (24)	0.92 (1)	0.36 (3)	0.57 (21)	0.32 (22)	0.87 (6)	0.21 (22)	0.91 (23)	0.92 (17)	0.55 (7)	0.53 (13)	0.70 (22)
25	0.94 (22)	0.82 (9)	0.92 (14)	0.43 (4)	0.57 (1)	0.29 (18)	0.87 (13)	0.24 (4)	0.90 (23)	0.93 (3)	0.50 (4)	0.75 (21)	0.74 (4)
26	0.94 (11)	0.81 (14)	0.92 (18)	0.37 (25)	0.57 (8)	0.29 (4)	0.87 (9)	0.14 (20)	0.90 (6)	0.93 (3)	0.55 (2)	0.74 (13)	0.91 (7)
27	0.93 (22)	0.80 (21)	0.92 (9)	0.68 (15)	0.57 (11)	0.28 (13)	0.87 (27)	0.14 (5)	0.91 (24)	0.93 (6)	0.54 (3)	0.74 (18)	0.89 (7)
28	0.94 (21)	0.47 (11)	0.92 (25)	0.65 (13)	0.56 (24)	0.87 (6)	0.86 (1)	0.65 (6)	0.91 (3)	0.93 (14)	0.51 (17)	0.73 (18)	0.89 (20)
29	0.94 (1)	0.81 (7)	0.92 (15)	0.64 (25)	0.55 (19)	0.86 (8)	0.86 (29)	0.84 (23)	0.91 (24)	0.92 (10)	0.51 (28)	0.72 (4)	0.88 (11)
30	0.86 (27)	0.81 (4)	0.92 (19)	0.64 (6)	0.52 (3)	0.79 (7)	0.81 (8)	0.69 (7)	0.91 (28)	0.93 (18)	0.51 (20)	0.73 (12)	0.88 (11)
31	0.92 (19)	0.81 (3)	0.92 (8)	0.64 (13)	0.51 (2)	0.84 (15)	0.85 (27)	0.83 (1)	0.90 (24)	0.93 (25)	0.50 (20)	0.72 (22)	0.87 (4)
32	0.90 (31)	0.80 (30)	0.92 (16)	0.63 (1)	0.56 (3)	0.67 (28)	0.85 (20)	0.63 (27)	0.88 (26)	0.93 (26)	0.49 (11)	0.72 (12)	0.87 (7)
33	0.85 (5)	0.66 (24)	0.92 (1)	0.62 (14)	0.47 (19)	0.82 (12)	0.85 (16)	0.75 (15)	0.91 (8)	0.92 (10)	0.35 (27,28)	0.71 (33)	0.87 (4)
34	0.93 (15)	0.81 (8)	0.92 (31)	0.63 (25)	0.55 (14)	0.45 (13)	0.84 (16)	0.74 (6)	0.90 (32)	0.92 (33)	0.47 (21)	0.72 (20)	0.86 (17)
35	0.86 (17)	0.47 (2)	0.92 (12)	0.62 (18)	0.49 (1)	0.72 (34)	0.84 (31)	0.78 (22)	0.90 (8)	0.92 (11)	0.48 (24)	0.71 (28)	0.87 (27)
36	0.85 (9)	0.80 (8)	0.92 (7)	0.62 (17)	0.49 (32)	0.74 (24)	0.62 (29)	0.72 (12)	0.90 (20)	0.92 (31)	0.46 (6)	0.72 (36)	0.86 (34)
37	0.85 (14)	0.39 (16)	0.92 (34)	0.61 (24)	0.49 (5)	0.74 (10)	0.83 (25)	0.83 (29)	0.90 (3)	0.92 (20)	0.44 (7)	0.71 (2)	0.86 (4)
38	0.86 (15)	0.80 (6)	0.92 (20)	0.61 (27)	0.48 (5)	0.62 (10)	0.83 (36)	0.53 (26)	0.91 (29)	0.92 (32)	0.46 (16)	0.71 (19)	0.85 (37)
39	0.86 (11)	0.39 (21)	0.92 (28)	0.61 (7)	0.54 (20)	0.78 (17)	0.84 (38)	0.56 (23)	0.90 (14)	0.92 (30)	0.32 (3)	0.71 (32)	0.86 (31)
40	0.85 (12)	0.71 (37)	0.92 (29)	0.61 (7)	0.56 (13)	0.75 (8)	0.83 (21)	0.82 (17)	0.90 (30)	0.92 (10)	0.46 (20)	0.71 (11)	0.86 (32)
41	0.86 (7)	0.76 (26)	0.92 (33)	0.61 (15)	0.49 (38)	0.49 (34)	0.82 (21)	0.51 (32)	0.90 (35)	0.92 (19)	0.44 (39)	0.71 (9)	0.85 (41)
42	0.85 (12)	0.61 (41)	0.92 (36)	0.61 (30)	0.48 (25)	0.78 (1)	0.82 (17)	0.80 (13)	0.89 (8)	0.92 (3)	0.42 (27)	0.70 (18)	0.85 (28)
43	0.92 (1)	0.69 (34)	0.92 (33)	0.61 (39)	0.49 (43)	0.70 (42)	0.81 (36)	0.49 (30)	0.90 (25)	0.92 (4)	0.43 (12)	0.71 (10)	0.85 (41)
44	0.84 (6)	0.63 (12)	0.91 (19)	0.61 (14)	0.56 (5)	0.65 (25)	0.82 (41)	0.71 (4)	0.90 (7)	0.92 (34)	0.41 (2)	0.71 (18)	0.85 (39)
45	0.84 (1)	0.75 (17)	0.91 (39)	0.60 (28)	0.48 (15)	0.70 (41)	0.81 (25)	0.60 (33)	0.90 (6)	0.92 (8)	0.33 (26)	0.70 (14)	0.83 (43)

Table S1 (continued)

K	Puerto Rican Paso Fino	Quarter Horse	Saddlebred	Shetland	Shire	Standardbred - Norway	Standardbred - US	Swiss Warmblood	Tennessee Walking Horse	Thoroughbred - UK	Thoroughbred - US	Tuva
2	0.61 (1)	0.69 (2)	0.65 (2)	0.97 (1)	0.89 (1)	0.71 (2)	0.70 (2)	0.78 (2)	0.62 (2)	1 (2)	1 (2)	0.67 (1)
3	0.53 (1)	0.68 (2)	0.64 (2)	0.99 (1)	0.75 (3)	0.70 (2)	0.69 (2)	0.77 (2)	0.61 (2)	0.99 (2)	0.99 (2)	0.57 (1)
4	0.76 (2)	0.51 (3)	0.54 (2)	0.98 (4)	0.74 (1)	0.59 (2)	0.57 (2)	0.63 (3)	0.53 (2)	0.99 (3)	0.99 (3)	0.45 (2)
5	0.81 (5)	0.48 (2)	0.42 (5)	0.98 (3)	0.74 (4)	0.90 (1)	0.83 (1)	0.61 (2)	0.38 (5)	0.99 (2)	0.99 (2)	0.49 (5)
6	0.73 (6)	0.49 (3)	0.57 (6)	0.98 (2)	0.66 (5)	0.65 (6)	0.63 (6)	0.62 (3)	0.56 (6)	0.99 (3)	0.99 (3)	0.38 (6)
7	0.56 (3)	0.50 (7)	0.31 (7)	0.98 (4)	0.67 (5)	0.90 (2)	0.83 (2)	0.63 (7)	0.27 (2,7)	0.99 (7)	0.99 (7)	0.46 (6)
8	0.76 (2)	0.47 (8)	0.49 (2)	0.98 (1)	0.66 (7)	0.90 (3)	0.82 (3)	0.61 (8)	0.44 (2)	0.99 (8)	0.99 (8)	0.40 (2)
9	0.72 (2)	0.40 (8)	0.93 (1)	0.98 (3)	0.66 (7)	0.89 (6)	0.79 (6)	0.57 (8)	0.93 (1)	0.99 (8)	0.99 (8)	0.34 (5)
10	0.72 (4)	0.41 (6)	0.91 (7)	0.97 (2)	0.66 (9)	0.89 (8)	0.79 (8)	0.57 (6)	0.92 (7)	0.99 (6)	0.99 (6)	0.29 (4)
11	0.72 (4)	0.41 (9)	0.94 (2)	0.97 (1)	0.66 (7)	0.89 (3)	0.79 (3)	0.57 (9)	0.87 (8)	0.99 (9)	0.99 (9)	0.30 (4)
12	0.87 (10)	0.41 (2)	0.95 (7)	0.97 (1)	0.66 (12)	0.89 (11)	0.79 (11)	0.57 (2)	0.88 (6)	0.99 (2)	0.99 (2)	0.26 (5)
13	0.83 (5)	0.41 (6)	0.95 (4)	0.97 (3)	0.66 (2)	0.89 (13)	0.79 (13)	0.57 (6)	0.88 (11)	0.99 (6)	0.99 (6)	0.28 (1)
14	0.83 (14)	0.41 (2)	0.94 (7)	0.97 (10)	0.66 (12)	0.89 (4)	0.79 (4)	0.57 (2)	0.87 (5)	0.99 (2)	0.99 (2)	0.22 (9)
15	0.82 (12)	0.42 (8)	0.90 (1)	0.97 (15)	0.62 (7)	0.89 (4)	0.79 (4)	0.58 (8)	0.87 (3)	0.99 (8)	0.99 (8)	0.28 (9)
16	0.83 (2)	0.42 (11)	0.91 (4)	0.97 (14)	0.63 (10)	0.88 (8)	0.79 (8)	0.58 (11)	0.87 (13)	0.99 (11)	0.99 (11)	0.17 (7)
17	0.83 (10)	0.42 (2)	0.91 (12)	0.94 (4)	0.63 (5)	0.89 (14)	0.79 (14)	0.58 (2)	0.87 (16)	0.99 (2)	0.99 (2)	0.18 (1)
18	0.81 (15)	0.42 (10,18)	0.92 (12)	0.98 (9)	0.63 (16)	0.89 (13)	0.80 (13)	0.58 (18)	0.87 (3)	0.99 (18)	0.99 (18)	0.62 (10)
19	0.81 (13)	0.49 (7)	0.90 (1)	0.94 (18)	0.63 (6)	0.90 (17)	0.80 (17)	0.57 (5)	0.88 (10)	0.99 (5)	0.99 (5)	0.56 (7)
20	0.82 (4)	0.43 (7)	0.91 (8)	0.94 91)	0.63 (19)	0.89 (9)	0.80 (9)	0.58 (7)	0.87 (18)	0.99 (7)	0.99 (7)	0.58 (11)
21	0.82 (6)	0.55 (5)	0.90 (7)	0.95 (9)	0.63 (15)	0.89 (18)	0.80 (18)	0.57 (14)	0.87 (10)	0.99 (14)	0.99 (14)	0.40 (2)
22	0.81 (2)	0.54 (20)	0.89 (11)	0.95 (18)	0.63 (14)	0.89 (1)	0.80 (1)	0.57 (17)	0.88 (9)	0.99 (17)	0.99 (17)	0.28 (20)
23	0.80 (16)	0.65 (13)	0.89 (14)	0.96 (1)	0.64 (5)	0.85 (15)	0.78 (15)	0.52 (8)	0.88 (7)	0.99 (8)	0.98 (8)	0.42 (11)
24	0.82 (14)	0.65 (7)	0.90 (12)	0.95 (21)	0.64 (20)	0.85 (16)	0.78 (16)	0.52 (3)	0.88 (9)	0.99 (3)	0.98 (3)	0.29 (22)
25	0.81 (6)	0.57 (4)	0.90 (12)	0.95 (1)	0.63 (11)	0.86 (10)	0.78 (10)	0.53 (15)	0.88 (5)	0.99 (15)	0.98 (15)	0.23 (18)
26	0.81 (5)	0.65 (2)	0.89 (1)	0.95 (8)	0.63 (15)	0.85 (22)	0.77 (22)	0.52 (25)	0.88 (23)	0.99 (25)	0.98 (25)	0.24 (4)
27	0.81 (1)	0.64 (3)	0.89 (19)	0.95 (11)	0.63 (8)	0.85 (12)	0.78 (12)	0.51 (10)	0.87 (25)	0.99 (10)	0.98 (10)	0.22 (13)
28	0.80 (10)	0.61 (17)	0.89 (16)	0.95 (24)	0.64 (27)	0.86 (22)	0.78 (22)	0.52 (26)	0.88 (15)	0.99 (26)	0.98 (26)	0.81 (6)
29	0.80 (12)	0.62 (28)	0.89 (14)	0.95 (19)	0.64 (9)	0.85 (6)	0.78 (6)	0.52 (13)	0.87 (16)	0.99 (13)	0.98 (13)	0.77 (8)
30	0.80 (14)	0.62 (20)	0.89 (17)	0.96 (3)	0.64 (21)	0.86 (25)	0.78 (25)	0.52 (30)	0.88 (23)	0.99 (30)	0.98 (30)	0.68 (7)
31	0.80 (16)	0.61 (20)	0.89 (23)	0.95 (2)	0.64 (6)	0.84 (26)	0.65 (26)	0.52 (12)	0.88 (31)	0.99 (12)	0.98 (12)	0.70 (15)
32	0.80 (9)	0.61 (11)	0.89 (23)	0.96 (3)	0.60 (13)	0.84 (21)	0.65 (21)	0.49 (17)	0.88 (19)	0.99 (17)	0.97 (17)	0.49 (6)
33	0.79 (21)	0.44 (28)	0.51 (30)	0.95 (19)	0.64 (26)	0.85 (23)	0.77 (23)	0.51 (27)	0.90 (30)	0.99 (27)	0.98 (27)	0.55 (12)
34	0.79 (10)	0.49 (21)	0.89 (9)	0.95 (14)	0.64 (22)	0.85 (26)	0.77 (26)	0.50 (5)	0.87 (11)	0.99 (5)	0.97 (5)	0.53 (4)
35	0.80 (23)	0.60 (24)	0.89 (15)	0.95 (1)	0.62 (10)	0.84 (29)	0.69 (29)	0.50 (19)	0.88 (13)	0.99 (19)	0.98 (19)	0.41 (34)
36	0.79 (2)	0.59 (6)	0.88 (3)	0.95 (32)	0.64 (15)	0.85 (11)	0.78 (11)	0.50 (18)	0.87 (16)	0.99 (18)	0.97 (18)	0.26 (24)
37	0.79 (31)	0.57 (7)	0.89 (18)	0.95 (5)	0.64 (1)	0.85 (32)	0.77 (32)	0.50 (21)	0.87 (12)	0.99 (21)	0.97 (21)	0.38 (10)
38	0.79 (1)	0.58 (16)	0.88 (23)	0.95 (5)	0.53 (28)	0.84 (30)	0.65 (30)	0.49 (4)	0.87 (38)	0.99 (4)	0.98 (4)	0.45 (10)
39	0.79 (1)	0.37 (16)	0.88 (9)	0.95 (20)	0.64 (4)	0.84 (6)	0.65 (6)	0.50 (3)	0.87 (15)	0.99 (3)	0.97 (3)	0.42 (17)
40	0.78 (4)	0.50 (20)	0.87 (33)	0.95 (13)	0.64 (38)	0.84 (22)	0.66 (22)	0.50 (1)	0.87 (16)	0.99 (1)	0.97 (1)	0.39 (8)
41	0.78 (29)	0.57 (39)	0.88 (3)	0.95 (38)	0.62 (24)	0.84 (23)	0.65 (23)	0.50 (6)	0.87 (40)	0.99 (6)	0.97 (6)	0.36 (4)
42	0.79 (6)	0.55 (27)	0.88 (35)	0.95 (25)	0.64 (2)	0.83 (4)	0.65 (4)	0.49 (24)	0.87 (39)	0.99 (24)	0.97 (24)	0.38 (1)
43	0.78 (22)	0.56 (12)	0.88 (13)	0.94 (43)	0.52 (26)	0.85 (17)	0.77 (17)	0.49 (7)	0.87 (19)	0.99 (7)	0.97 (7)	0.35 (42)
44	0.78 (21)	0.55 (2)	0.87 (22)	0.95 (5)	0.64 (17)	0.85 (29)	0.66 (29)	0.50 (1)	0.86 (3)	0.99 (1)	0.97 (1)	0.28 (25)
45	0.78 (20)	0.36 (44)	0.87 (21)	0.95 (15)	0.64 (45)	0.84 (10)	0.65 (10)	0.51 (26)	0.87 (4)	0.98 (26)	0.97 (26)	0.33 (41)
