# Supplementary material for: Genetic Diversity in the Modern Horse Illustrated from Genome-Wide SNP Data
Source: PLoS One. 2013 Jan 30;8(1):e54997. doi: 10.1371/journal.pone.0054997 (PMC3559798; doi:10.1371/journal.pone.0054997)
Supplement: Table S2 — Proportion of assignment for 38 horse populations to each of K = 29 clusters. Proportion of assignment to each of K = 29 clusters as determined in Structure. The largest proportion of assignment for each population is outlined and shown in bold; those with 30–50% assignment are shown in italic. The top row notes the breed(s) with >50% of assignment to each of the 29 clusters. This analysis was performed without removal of outlier individuals. (PDF) [file pone.0054997.s009.pdf]

Table S2. Proportion of assignment to each of K=29 clusters as determined in Structure. The largest proportion of assignment for each population is outlined and shown in bold; those with 30-50% assignment are shown in italic. The top row notes the breed(s) with > 50% of assignment to each of the 29 clusters. This analysis was performed without removal of outlier individuals.

**Population with > 50% assignment to the Cluster**

| Population | Icelandic | Akhal Teke | Exmoor | Percheron | Franches-Montagnes | Standardbred | Andalusian, Lusitano | Mongolian, Tuva | Clydesdale, Shire | Norwegian Fjord | Peruvian Paso | Puerto Rican Paso Fino | Hanoverian, Swiss Warmblood, Thoroughbred | Saddlebred | Mangalarga Paulista | Tennessee Walking Horse | Arabian | French Trotter | Miniature, Shetland | Fell Pony | Belgian | Finnhorse | New Forest Pony | North Swedish Horse | Maremmano | Florida Cracker | Caspian | Paint, Quarter Horse | Morgan |
|---|---|---|---|---|---|---|---|---|---|---|---|---|---|---|---|---|---|---|---|---|---|---|---|---|---|---|---|---|---|
| **Structure Cluster (K=29)** | 1 | 2 | 3 | 4 | 5 | 6 | 7 | 8 | 9 | 10 | 11 | 12 | 13 | 14 | 15 | 16 | 17 | 18 | 19 | 20 | 21 | 22 | 23 | 24 | 25 | 26 | 27 | 28 | 29 |
| Akhal Teke | 0.001 | **0.867** | 0.001 | 0.001 | 0.001 | 0.001 | 0.004 | 0.001 | 0 | 0.001 | 0.002 | 0.001 | 0.060 | 0.001 | 0.001 | 0.002 | 0.036 | 0.001 | 0.001 | 0.001 | 0.001 | 0.001 | 0.008 | 0.001 | 0.001 | 0.001 | 0.001 | 0.003 | 0.001 |
| Andalusian | 0.001 | 0.003 | 0.002 | 0.001 | 0.002 | 0.004 | **0.915** | 0.010 | 0.001 | 0.002 | 0.004 | 0.006 | 0.002 | 0.005 | 0.003 | 0.001 | 0.016 | 0.002 | 0 | 0.001 | 0.001 | 0.003 | 0.001 | 0.001 | 0.004 | 0.001 | 0.001 | 0.004 | 0.003 |
| Arabian | 0.001 | 0.005 | 0.001 | 0.001 | 0.001 | 0.003 | 0.002 | 0.004 | 0.001 | 0.001 | 0.004 | 0.001 | 0.023 | 0.001 | 0.002 | 0.002 | **0.930** | 0.002 | 0.001 | 0.001 | 0 | 0.001 | 0.002 | 0 | 0.002 | 0.001 | 0.002 | 0.004 | 0.002 |
| Belgian | 0.003 | 0.001 | 0.002 | 0.014 | 0.005 | 0.001 | 0.001 | 0.008 | 0.010 | 0.005 | 0.001 | 0.001 | 0.001 | 0.001 | 0.001 | 0.003 | 0.001 | 0.001 | 0.001 | 0.002 | **0.917** | 0.009 | 0.003 | 0.001 | 0.001 | 0.003 | 0.002 | 0.001 | 0.001 |
| Caspian | 0.003 | 0.011 | 0.001 | 0.001 | 0.004 | 0.001 | 0.003 | 0.068 | 0.002 | 0.002 | 0.015 | 0.001 | 0.006 | 0.019 | 0.002 | 0.001 | 0.068 | 0.001 | 0.001 | 0.004 | 0.003 | 0.002 | 0.024 | 0.001 | 0.003 | 0.003 | **0.737** | 0.007 | 0.003 |
| Clydesdale | 0 | 0 | 0 | 0.002 | 0.001 | 0.001 | 0 | 0 | **0.976** | 0.001 | 0 | 0 | 0 | 0 | 0 | 0 | 0 | 0 | 0 | 0.002 | 0.001 | 0 | 0.010 | 0 | 0 | 0 | 0 | 0.001 | 0.001 |
| Exmoor | 0.001 | 0 | **0.941** | 0.002 | 0.003 | 0 | 0.001 | 0.014 | 0.002 | 0.001 | 0.001 | 0.001 | 0.001 | 0.001 | 0.001 | 0 | 0.001 | 0.001 | 0.005 | 0.002 | 0.001 | 0.001 | 0.012 | 0 | 0.001 | 0.001 | 0.001 | 0.002 | 0.001 |
| Fell Pony | 0.002 | 0.001 | 0.002 | 0.002 | 0.006 | 0.001 | 0 | 0.002 | 0.103 | 0.001 | 0.001 | 0 | 0.001 | 0.001 | 0.001 | 0.001 | 0.001 | 0.001 | 0.001 | **0.851** | 0.001 | 0.003 | 0.009 | 0.004 | 0.001 | 0.001 | 0.001 | 0.001 | 0.001 |
| Finnhorse | 0.017 | 0.001 | 0.002 | 0.007 | 0.004 | 0.001 | 0.002 | 0.035 | 0.005 | 0.008 | 0.002 | 0.001 | 0.001 | 0.001 | 0.002 | 0.001 | 0.001 | 0.001 | 0.005 | 0.004 | 0.004 | **0.865** | 0.011 | 0.013 | 0.002 | 0.001 | 0.002 | 0.001 | 0.002 |
| Florida Cracker | 0.004 | 0.006 | 0.001 | 0.001 | 0.001 | 0.006 | 0.004 | 0.033 | 0.002 | 0.002 | 0.003 | 0 | 0.042 | 0.023 | 0.001 | 0.002 | 0.036 | 0.001 | 0.001 | 0.004 | 0.001 | 0.001 | 0.014 | 0.001 | 0.005 | **0.723** | 0.001 | 0.079 | 0.005 |
| Franches-Montagnes | 0.001 | 0.001 | 0.001 | 0.005 | **0.887** | 0.001 | 0.001 | 0.001 | 0.001 | 0.001 | 0.001 | 0.001 | 0.046 | 0.001 | 0.001 | 0.001 | 0.002 | 0.001 | 0.001 | 0.001 | 0.031 | 0.003 | 0.003 | 0.001 | 0.002 | 0.002 | 0.001 | 0.002 | 0.001 |
| French Trotter | 0.001 | 0.001 | 0.002 | 0.001 | 0.001 | 0.042 | 0.001 | 0.001 | 0.001 | 0 | 0.001 | 0.001 | 0.161 | 0.002 | 0.001 | 0.002 | 0.005 | **0.759** | 0.001 | 0.001 | 0.001 | 0.002 | 0.002 | 0.001 | 0.001 | 0.001 | 0.001 | 0.006 | 0.003 |
| Hanoverian | 0.001 | 0.008 | 0.001 | 0.005 | 0.023 | 0.004 | 0.004 | 0.012 | 0.003 | 0.002 | 0.005 | 0.002 | **0.514** | 0.015 | 0.003 | 0.001 | 0.024 | 0.025 | 0.001 | 0.003 | 0.001 | 0.002 | 0.194 | 0.002 | 0.040 | 0.002 | 0.011 | 0.078 | 0.014 |
| Icelandic | **0.937** | 0.001 | 0.001 | 0.002 | 0.001 | 0.001 | 0.001 | 0.003 | 0.001 | 0.004 | 0.001 | 0.001 | 0 | 0.001 | 0.001 | 0 | 0 | 0.001 | 0.028 | 0.001 | 0.002 | 0.003 | 0.001 | 0.005 | 0.001 | 0.001 | 0.001 | 0 | 0.001 |
| Lusitano | 0.004 | 0.002 | 0.001 | 0.001 | 0.002 | 0.001 | **0.808** | 0.005 | 0.002 | 0.001 | 0.049 | 0.006 | 0.008 | 0.004 | 0.002 | 0.001 | 0.072 | 0.002 | 0.001 | 0.001 | 0.001 | 0.003 | 0.003 | 0.001 | 0.004 | 0.002 | 0.007 | 0.003 | 0.002 |
| Mangalarga Paulista | 0.002 | 0.003 | 0 | 0.001 | 0.001 | 0.001 | 0.003 | 0.006 | 0 | 0.001 | 0.011 | 0.001 | 0.026 | 0.001 | **0.920** | 0.001 | 0.001 | 0.005 | 0 | 0 | 0.001 | 0.001 | 0.002 | 0.001 | 0.003 | 0.001 | 0.001 | 0.005 | 0.002 |
| Maremmano | 0.002 | 0.003 | 0.002 | 0.004 | 0.003 | 0.003 | 0.003 | 0.008 | 0.004 | 0.002 | 0.003 | 0.002 | 0.262 | 0.003 | 0.002 | 0.001 | 0.002 | 0.007 | 0.002 | 0.002 | 0.004 | 0.004 | 0.015 | 0.002 | **0.642** | 0.001 | 0.005 | 0.004 | 0.004 |
| Miniature | 0.112 | 0.001 | 0.002 | 0.001 | 0.003 | 0.003 | 0.004 | 0.068 | 0.002 | 0.003 | 0.001 | 0.001 | 0.005 | 0.001 | 0.001 | 0.001 | 0.001 | 0.003 | **0.549** | 0.005 | 0.002 | 0.022 | 0.197 | 0.002 | 0.005 | 0.002 | 0.001 | 0.001 | 0.001 |
| Mongolian | 0.029 | 0.001 | 0.001 | 0.002 | 0.004 | 0.001 | 0.001 | **0.855** | 0.001 | 0.011 | 0.001 | 0.003 | 0.020 | 0 | 0.001 | 0.001 | 0 | 0.001 | 0.014 | 0.004 | 0.006 | 0.019 | 0.007 | 0.006 | 0.002 | 0.003 | 0.005 | 0.001 | 0.001 |
| Morgan | 0.002 | 0.004 | 0.001 | 0.003 | 0.001 | 0.007 | 0.004 | 0.013 | 0.002 | 0.001 | 0.005 | 0.003 | 0.009 | 0.021 | 0.001 | 0.005 | 0.006 | 0.008 | 0.001 | 0.002 | 0.003 | 0.002 | 0.011 | 0.001 | 0.006 | 0.002 | 0.003 | 0.013 | **0.860** |
| New Forest Pony | 0.003 | 0.004 | 0.015 | 0.005 | 0.004 | 0.003 | 0.002 | 0.015 | 0.008 | 0.003 | 0.004 | 0.002 | 0.006 | 0.002 | 0.001 | 0.001 | 0.011 | 0.002 | 0.008 | 0.045 | 0.007 | 0.002 | **0.836** | 0.001 | 0.004 | 0.002 | 0.002 | 0.002 | 0.002 |
| North Swedish Horse | 0.005 | 0.001 | 0.002 | 0.003 | 0.003 | 0.001 | 0.001 | 0.011 | 0.001 | 0.020 | 0.002 | 0.001 | 0.001 | 0.001 | 0.002 | 0.001 | 0.001 | 0.001 | 0.005 | 0.002 | 0.007 | 0.009 | 0.008 | **0.909** | 0.001 | 0.001 | 0.002 | 0.001 | 0 |
| Norwegian Fjord | 0.019 | 0 | 0.002 | 0.001 | 0.001 | 0.001 | 0.001 | 0.001 | 0.001 | **0.924** | 0.001 | 0 | 0 | 0.001 | 0.001 | 0.001 | 0.001 | 0.001 | 0.009 | 0.001 | 0.004 | 0.015 | 0.002 | 0.010 | 0.001 | 0 | 0 | 0.001 | 0.001 |
| Paint | 0.002 | 0.005 | 0.001 | 0.002 | 0.004 | 0.007 | 0.003 | 0.017 | 0.002 | 0.002 | 0.014 | 0.002 | *0.340* | 0.013 | 0.001 | 0.014 | 0.005 | 0.009 | 0.002 | 0.002 | 0.004 | 0.004 | 0.015 | 0.002 | 0.003 | 0.003 | 0.007 | **0.508** | 0.007 |
| Percheron | 0.003 | 0.001 | 0.001 | **0.722** | 0.005 | 0.001 | 0.001 | 0.004 | 0.005 | 0.003 | 0.002 | 0.001 | 0.001 | 0.001 | 0.001 | 0.001 | 0 | 0.001 | 0.001 | 0.001 | 0.228 | 0.002 | 0.003 | 0.003 | 0.002 | 0.003 | 0.001 | 0.001 | 0.004 |
| Peruvian Paso | 0.001 | 0.005 | 0.001 | 0.001 | 0.001 | 0.001 | 0.039 | 0.002 | 0.001 | 0.002 | **0.881** | 0.016 | 0.006 | 0.001 | 0.006 | 0.001 | 0.004 | 0.002 | 0.002 | 0.001 | 0.001 | 0.001 | 0.002 | 0.002 | 0.008 | 0.003 | 0.003 | 0.004 | 0.003 |
| Puerto Rican Paso Fino | 0.001 | 0.003 | 0.004 | 0.002 | 0.001 | 0.002 | 0.029 | 0.051 | 0.001 | 0.002 | 0.042 | **0.802** | 0.002 | 0.001 | 0.008 | 0.001 | 0.008 | 0.002 | 0.003 | 0.001 | 0.003 | 0.003 | 0.009 | 0.002 | 0.004 | 0.004 | 0.004 | 0.003 | 0.002 |
| Quarter Horse | 0.001 | 0.002 | 0.001 | 0.004 | 0.002 | 0.001 | 0.005 | 0.003 | 0.001 | 0.001 | 0.006 | 0.001 | *0.301* | 0.006 | 0.001 | 0.004 | 0.008 | 0.003 | 0.002 | 0.001 | 0.002 | 0.002 | 0.006 | 0.001 | 0.002 | 0.004 | 0.004 | **0.616** | 0.007 |
| Saddlebred | 0.001 | 0.007 | 0.001 | 0.001 | 0.002 | 0.012 | 0.002 | 0.002 | 0.001 | 0.001 | 0.003 | 0.001 | 0.015 | **0.888** | 0.001 | 0.002 | 0.006 | 0.009 | 0.001 | 0.001 | 0.001 | 0.002 | 0.002 | 0.001 | 0.003 | 0.003 | 0.001 | 0.016 | 0.016 |
| Shetland | 0.019 | 0.001 | 0.002 | 0.002 | 0.001 | 0.001 | 0 | 0.003 | 0.001 | 0.003 | 0.001 | 0.001 | 0 | 0 | 0 | 0.001 | 0 | 0.001 | **0.954** | 0.001 | 0.001 | 0.003 | 0.003 | 0.001 | 0 | 0.001 | 0.001 | 0.001 | 0.001 |
| Shire | 0.001 | 0.001 | 0.001 | 0.030 | 0.003 | 0.001 | 0.001 | 0.008 | **0.640** | 0.003 | 0.001 | 0.001 | 0.048 | 0.003 | 0.001 | 0.002 | 0.020 | 0.001 | 0.001 | 0.060 | 0.077 | 0.003 | 0.068 | 0.003 | 0.005 | 0.001 | 0.004 | 0.012 | 0.001 |
| Standardbred - Norway | 0.001 | 0.001 | 0.001 | 0.001 | 0.001 | **0.853** | 0.001 | 0.003 | 0.001 | 0 | 0.001 | 0.001 | 0.009 | 0.008 | 0.001 | 0.003 | 0.003 | 0.100 | 0 | 0.001 | 0.001 | 0.001 | 0.001 | 0.001 | 0.001 | 0.001 | 0.001 | 0.003 | 0.001 |
| Standardbred - US | 0.001 | 0.001 | 0 | 0.001 | 0.001 | **0.776** | 0.002 | 0.006 | 0 | 0 | 0.001 | 0 | 0.025 | 0.012 | 0.001 | 0.026 | 0.002 | 0.025 | 0 | 0.003 | 0.001 | 0.003 | 0.014 | 0 | 0.002 | 0.001 | 0.001 | 0.079 | 0.015 |
| Swiss Warmblood | 0.001 | 0.013 | 0.003 | 0.015 | 0.046 | 0.002 | 0.004 | 0.009 | 0.001 | 0.001 | 0.002 | 0.005 | **0.515** | 0.010 | 0.001 | 0.012 | 0.028 | 0.095 | 0.001 | 0.001 | 0.006 | 0.003 | 0.100 | 0.002 | 0.028 | 0.005 | 0.005 | 0.073 | 0.012 |
| Tennessee Walking Horse | 0.001 | 0.004 | 0.001 | 0.001 | 0.004 | 0.013 | 0.002 | 0.002 | 0.001 | 0.001 | 0.001 | 0.001 | 0.014 | 0.018 | 0.001 | **0.871** | 0.003 | 0.004 | 0.001 | 0.002 | 0.001 | 0.001 | 0.016 | 0.002 | 0.003 | 0.001 | 0.001 | 0.025 | 0.007 |
| Thoroughbred - UK/Ire | 0 | 0 | 0 | 0 | 0 | 0.001 | 0 | 0 | 0 | 0 | 0 | 0 | **0.990** | 0.001 | 0 | 0.001 | 0 | 0 | 0 | 0 | 0 | 0 | 0 | 0 | 0 | 0 | 0 | 0 | 0.001 |
| Thoroughbred - US | 0 | 0.001 | 0 | 0 | 0 | 0.002 | 0.001 | 0.001 | 0 | 0 | 0 | 0.001 | **0.978** | 0.002 | 0 | 0.001 | 0.001 | 0.002 | 0 | 0 | 0 | 0.001 | 0.001 | 0 | 0.001 | 0.001 | 0.001 | 0.003 | 0.001 |
| Tuva | 0.004 | 0.014 | 0.003 | 0.007 | 0.007 | 0.006 | 0.007 | **0.767** | 0.004 | 0.009 | 0.018 | 0.002 | 0.017 | 0.002 | 0.001 | 0.005 | 0.023 | 0.003 | 0.005 | 0.003 | 0.011 | 0.019 | 0.014 | 0.003 | 0.003 | 0.003 | 0.005 | 0.011 | 0.026 |
